# Spatiotemporally Guided Single‐Atom Bionanozyme for Targeted Antibiofilm Treatment

**DOI:** 10.1002/smll.202407747

**Published:** 2024-10-06

**Authors:** Lunjie Huang, Hongbin Pu, Da‐Wen Sun

**Affiliations:** ^1^ School of Food Science and Engineering South China University of Technology Guangzhou 510641 China; ^2^ Academy of Contemporary Food Engineering South China University of Technology Guangzhou Higher Education Mega Centre Guangzhou 510006 China; ^3^ Engineering and Technological Research Centre of Guangdong Province on Intelligent Sensing and Process Control of Cold Chain Foods & Guangdong Province Engineering Laboratory for Intelligent Cold Chain Logistics Equipment for Agricultural Products Guangzhou Higher Education Mega Centre Guangzhou 510006 China; ^4^ Food Refrigeration and Computerized Food Technology (FRCFT) Agriculture and Food Science Centre University College Dublin National University of Ireland Belfield Dublin D04 V1W8 Ireland

**Keywords:** antibacterial, biofilm microenvironment, MOFs, nanobiohybrids, nanozyme

## Abstract

The heterogeneous and dynamic microenvironment of biofilms complicates bacterial infection treatment. Nanozyme catalytic therapy has recently been promising in treating biofilm infections. However, active nanozymes designed with the required precision targeting the biofilm microenvironment are lacking. This work proposes a spatiotemporally guided single‐atom bionanozyme (BioSAzyme) for targeted antibiofilm therapy based on protein engineering of copper single‐atom nanozyme (Cu SAzyme). The Cu SAzyme, synthesized via a novel mechanochemistry‐assisted method, features highly accessible Cu–N_4_ active sites exposed on 2D N‐doped carbon, exhibiting excellent triple enzyme‐like activities according to experimental results and density functional theory calculations. Inheriting biofunctionality from both glucose oxidase and concanavalin A, BioSAzyme can localize the biofilm glycocalyx and catalyze endogenous glucose into H₂O₂ and gluconic acid, thus triggering multiplex cascade reactions with pH self‐adaption to consume glucose and glutathione and generate •OH radicals. This spatiotemporally guided bionanocatalytic agent effectively inhibits *E. coli* O157: H7 and methicillin‐resistant *S. aureus* biofilms in vitro and in vivo. Taking together, this work opens up new avenues for the rational design of single‐atom nanozymes for precise antibiofilm therapy.

## Graphical abstract

BioSAzyme, a novel 2D copper single‐atom bionanozyme, integrates multi‐enzyme‐like Cu‐N_4_ sites with the biorecognizable GOX and ConA proteins, initiating adaptive cascade reactions that localize to the biofilm glycocalyx and target metabolic environments. This innovative protein‐directed nanobiohybrid enables spatiotemporally precise eradication of *E. coli* and MRSA biofilms both in vitro and in vivo, paving the way for advanced antibiofilm strategies.

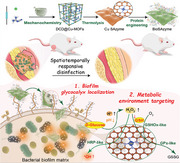

## 1 Introduction

Biofilms are often associated with human bacterial infections and the resulting antibiotic resistance. Unlike planktonic bacteria, bacterial biofilms create a highly heterogeneous and compartmentalized microenvironment by embedding bacterial aggregates within extracellular polymeric substances (EPS), forming a robust barrier against antibiotics and immune cells.^[^ ^1^ ^]^ The EPS matrix physically isolates antibacterial molecules, limiting their penetration through processes such as chelation and enzymatic degradation. Additionally, the biofilm microenvironment exhibits chemical and physiological heterogeneity, including hypoxia, nutrient limitation, pH partitioning, and glutathione (GSH) overexpression, which facilitate bacterial survival, adaptation, and persistent infection.^[^ ^2^ ^]^ Therefore, exploiting the unique microenvironment of biofilms to disrupt their homeostasis represents a promising strategy for preventing biofilm infections.^[^ ^3^ ^]^

Nanozyme catalytic therapy, which utilizes enzyme‐like nanomaterials (nanozymes) for tunable biochemical reactions, has shown potential for enhancing the precision and efficacy of biofilm treatments.^[^ ^4^ ^]^ For instance, various nanozymes, such as V_2_O_5_,^[^ ^5^ ^]^ MoS_2_,^[^ ^6^ ^]^ CeO_2_,^[^ ^7^ ^]^ FeS,^[^ ^8^ ^]^ and Fe_3_O_4_,^[^ ^9^ ^]^ have been explored for anti‐biofilm applications.^[^ ^10^ ^]^ However, most current nanomaterials lack the fine structures to mimic the complexity of enzymes, resulting in suboptimal functional selectivity and activity.^[^ ^11^ ^]^ In contrast, the intricate protein skeletons of natural enzymes, often embedded with single‐atom metal active centers^[^ ^12^ ^]^—like the single Fe site in hemoglobin, the single Mg site in chlorophyll, and the single Mo site in nitrogenase—provide the structural basis for their high selectivity and efficiency. Single‐atom catalysts (SACs), which contain isolated single atoms as catalytic centers, are considered the ultimate design for precise catalysts due to their unique and homogeneous structures, allowing for the detailed elucidation of catalytic mechanisms and the customization of single‐atom nanozymes (SAzymes).^[^ ^13^ ^]^ SACs combine the advantages of heterogeneous and homogeneous catalysts, offering exceptional activity, selectivity, maximum atom utilization efficiency, low cost, and renewability. Metal‐organic frameworks (MOFs) have emerged as a reliable toolbox for constructing SAzymes for bio‐applications,^[^ ^14^ ^]^ with well‐defined local structures and strong interactions between substrates and catalytic active sites. For example, Dong group^[^ ^12b^ ^]^ reported oxidase‐like single‐atom nanozymes derived from ZIF‐8, featuring carbon nanoframe‐confined FeN_5_ active centers, which showed significantly enhanced antibacterial activity both in vitro and in vivo. However, their availability is limited to a few solvothermally synthesized MOFs, such as ZIF‐8 and Zr‐TCPP.^[^ ^15^ ^]^ Thus, continued exploration is needed toward greener synthesis methods and performance enhancement.

Moreover, the rational engineering of single‐atom nanozymes to actively adapt to and disrupt biofilm microenvironments is of great interest. For instance, peroxidase‐ (POD) like nanozymes, as typical tools for reactive‐oxygen‐species (ROS) nanozyme therapy, exhibit POD‐like activity under acidic conditions (pH 3–6) by pre‐adsorbing H^+^ and catalytically decomposing H₂O₂, producing hydroxyl radicals (•OH) for bactericidal effects.^[^ ^16^ ^]^ Zhang et al.,^[^ ^17^ ^]^ for instance, developed a therapy for the targeted eradication of resistant *Helicobacter pylori* through the in vivo activation of a cascade nanozyme with acid pH‐responsive nanocatalytic oxygenation. However, the pH microenvironment of infectious biofilms is often variable or near‐neutral,^[^ ^18^ ^]^ potentially rendering nanozyme therapy ineffective. Additionally, the endogenous H₂O₂ levels in biofilms are insufficient for effective antibacterial action, and introducing exogenous H₂O₂ poses operational and safety concerns. Researchers have identified glucose oxidase (GOX) as an effective enzyme that can convert endogenous glucose into H₂O₂ under physiological conditions, spontaneously initiating biofilm‐responsive cascade therapeutic reactions.^[^ ^19^ ^]^ Furthermore, bacteria within biofilms can reduce ROS levels and evade oxidative damage through overexpressed GSH,^[^ ^20^ ^]^ conferring high resistance or tolerance to ROS‐based nanozyme therapy.^[^ ^21^ ^]^ Yang et al.^[^ ^22^ ^]^ demonstrated that selenium‐ and chlorin‐containing polydopamine nanozyme not only depletes endogenous GSH to enhance the anti‐biofilm effects of reactive species but also degrades GSH through cascade reactions, generating more lethal reactive species for effective biofilm eradication. Therefore, GSH depletion significantly contributes to the targeted antibiofilm activity of nanozymes.

**Scheme 1:**
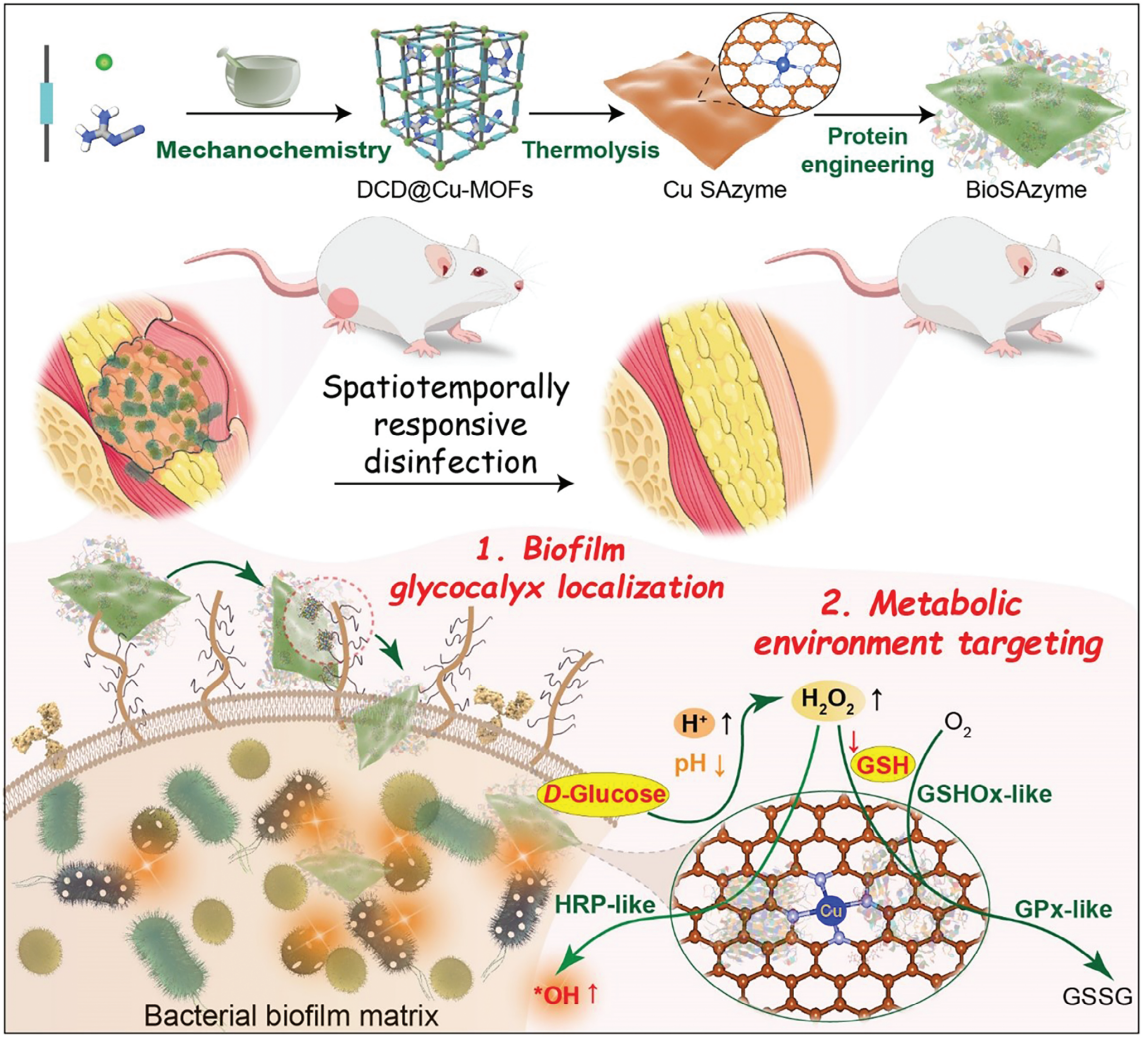
Protein‐directed single‐atom bionanocatalysis enables biofilm‐responsive parallel catalytic disinfection with spatiotemporal precision.

Importantly, an effective prerequisite for combating biofilms through these parallel enzyme‐mimicking reactions is the sufficient recognition and strong binding between nanozymes and biofilms. This interaction is crucial for overcoming the spatial barrier effect posed by the biofilm's EPS matrix.^[^ ^23^ ^]^ Typical nanozyme therapies often rely on passive penetration strategies, such as reducing nanoparticle size or employing biomimetic structures like virus synapses and needle‐like designs.^[^ ^24^ ^]^ In contrast, recent nanomedicines emphasize the significance of conjugating affinity‐binding bioentities to the nanoparticle surface for therapeutic efficacy.^[^ ^25^ ^]^ For instance, soluble lectins or adhesin proteins like concanavalin A (ConA) exhibit bioaffinity toward the glycocalyx of EPS, and are often used to enhance the localization of nanodrugs at the periphery of the biofilm matrix.^[^ ^26^ ^]^ However, the importance of grafting surfaces with bioaffinity entities has been overlooked in single‐atom catalytic therapy. To address these challenges, this study employs a solvent‐free mechanochemical derivatization route to synthesize a 2D copper single‐atom nanozyme (Cu SAzyme) with highly accessible active sites and multiple enzyme‐like activities. Through post‐synthetic protein engineering, the resulting single‐atom bionanohybrid (BioSAzyme) is endowed with biofilm bioaffinity and cascade reactivity, which enhance the spatiotemporal precision and efficacy of single‐atom nanozyme‐based antibiofilm therapies (**Scheme** **1**), showing great potential for the targeted eradication of infectious biofilms.

## 2 Results and Discussion

### 2.1 Preparation and Characterization of Cu SAzymes

**Figure 1:**
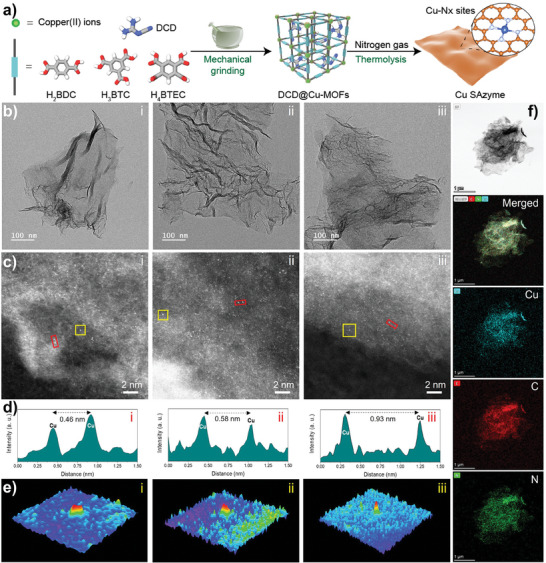
a) The synthesis procedure, b) TEM images, and c) HAADF‐STEM images of Cu SAzymes. d) Line‐scanning intensity profiles of the distance between Cu atoms marked with a red rectangle in (c). e) Atom‐overlapping Gaussian‐function‐fitting mapping from the yellow squares in (c). (i‐iii) represents Cu SAC‐BDC, Cu SAC‐BTC, and Cu SAC‐BTEC samples, respectively. f) Elemental distribution mapping images collected for the SAzyme‐BDC sample. Scale bar is 1 µm.

Generally, synthesizing highly dispersed single‐atom nanozymes via MOF pyrolysis is limited by the internal nitrogen and metal content.^[^ ^14c^ ^]^ To address this, a green and straightforward mechanochemical method was employed to prepare three Cu‐MOFs (Cu‐BDC, Cu‐BTC, and Cu‐BTEC) using terephthalic acid (H_2_BDC), trimesic acid (H_3_BTC), and 1,2,4,5‐benzenetetracarboxylic acid (H_4_BTEC) as ligands. Dicyandiamide (DCD) served as a guest molecule in Cu‐MOFs to capture metal atoms, which is crucial for forming highly stable single‐atom nanozymes (**Figure** **1**a). Scanning electron microscopy (SEM) images of Cu‐MOFs prepared by mechanochemistry with and without DCD are shown in Figure S1 (Supporting Information). The results indicate that Cu‐BDC, Cu‐BTC, and Cu‐BTEC exhibit distinct morphologies: cotton‐like, rod‐like, and pyramidal structures, respectively. When DCD was added during the mechanochemical reaction, Cu‐BDC@DCD, Cu‐BTC@DCD, and Cu‐BTEC@DCD all exhibited 2D sheet‐like morphologies, laying an essential foundation for the well‐exposed single metal sites in the Cu SAC structure.

After pyrolysis and acid washing, the Cu‐MOFs@DCD precursors were converted into the desired Cu SAC structures. High‐resolution transmission electron microscopy (HR‐TEM) images of Cu SAC‐BDC i), Cu SAC‐BTC ii), and Cu SAC‐BTEC iii) samples (Figure 1b) showed semi‐transparent graphene‐like nanosheet structures, with no observable nanoparticle distribution, suggesting that the Cu sites in Cu SAC samples are likely single‐atom dispersed and highly accessible. To confirm the single‐atom species of Cu, aberration‐corrected high‐angle annular dark‐field scanning transmission electron microscopy (HAADF‐STEM) was used for close observation of these samples. As shown in Figure 1c, a large number of highly isolated copper atoms (small bright spots) are fixed on the amorphous carbon carrier in Cu SAC samples. Ordered graphene regions can also be observed in the HR‐TEM images of the nanosheets (Figure S2, Supporting Information). The intensity distribution (Figure 1d) obtained from the red rectangles in Figure 1c indicates that the distances between isolated Cu sites are 0.46, 0.58, and 0.93 nm, far greater than the Cu─Cu bond length. Additionally, the 3D atom‐overlapping Gaussian‐function fitting mapping (Figure 1e) of the yellow rectangle in Figure 1c reveals that heavier Cu atoms are uniformly decorated on the carbon layer. Elemental mapping of Cu SAC‐BDC further confirms the uniform distribution of Cu, C, and N elements in the 2D nanosheets (Figure 1f). These results verify that Cu elements in Cu SAC samples are likely dispersed as single atoms. Additionally, the Cu loading of Cu SAC‐BDC, Cu SAC‐BTC, and Cu SAC‐BTEC samples, obtained from inductively coupled plasma optical emission spectrometry (ICP‐OES), were 28.27%, 10.68%, and 19.80%, respectively.

**Figure 2:**
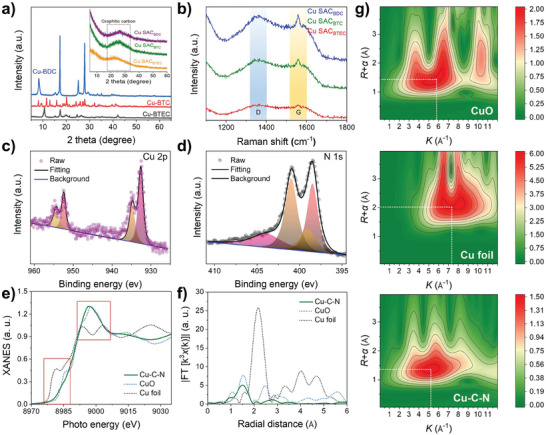
a) PXRD patterns and b) Raman spectra of the samples. c,d) XPS spectra at the Cu 2p and N 1s regions were collected for the SAzyme‐BDC sample, respectively. e) XANES spectra, f) Fourier transform EXAFS spectra at Cu K‐edge, and g) the corresponding Wavelet transforms for EXAFS signals of different samples.

Powder X‐ray diffraction (PXRD) was used to further study the crystalline properties of Cu SACs. As shown in **Figure** **2**a, the precursor Cu‐MOFs exhibited good crystallinity, whereas the Cu SAC samples only showed a broad peak, similar to the carbonized DCD and H_2_BDC mixture peak (Figure S3, Supporting Information), likely attributed to the graphite carbon (002) plane.^[^ ^27^ ^]^ Unlike the PXRD patterns of copper metal salts and DCD mixtures or Cu‐MOF derivatives, which show characteristic peaks corresponding to Cu, CuO, and Cu_2_O crystals (Figure S4, Supporting Information), no such impurity peaks were observed in Cu SAC samples. Raman spectra revealed the amorphous carbon properties with topological defects in Cu SACs (Figure 2b) and control samples (Figure S5, Supporting Information) (D, disorder defects; G, graphite phase).^[^ ^13b^ ^]^ These results support that Cu SAC samples are likely single‐atom dispersed.

Theoretically, high metal atom loading in single‐atom materials challenges their single‐atom dispersion state. Thus, subsequent characterizations focused on Cu SAC‐BDC, which had the highest Cu loading. X‐ray photoelectron spectroscopy (XPS) was employed to study the chemical states of Cu and N elements in the sample (Figure S6, Supporting Information). In the high‐resolution Cu 2p XPS spectrum (Figure 2c), two pairs of characteristic peaks appeared at ≈934.8/954.1 eV and 932.3/952.5 eV, which can be attributed to Cu^2^⁺ and [Cu⁰ + Cu⁺] valence states, respectively.^[^ ^28^ ^]^ In the high‐resolution N 1s spectrum (Figure 2d), four peaks at 398.6, 399.0, 400.9, and 403.9 eV can be attributed to pyridine, pyrrole, graphitic components, and oxidized states of N species, respectively.^[^ ^27^ ^]^ Therefore, Cu SACs exhibited typical Cu–N–C 2D structures. Nitrogen plays a key role in forming single‐atom Cu in Cu–N–C. During the growth process, heteroatom N strongly coordinates with Cu atoms, introducing spatial spacing to inhibit the “Ostwald ripening” of Cu atoms.^[^ ^27^ ^]^ Uniformly distributed heteroatom N also stabilizes Cu atoms in the carbon matrix, inducing stable and uniform distribution of Cu atoms in Cu–N–C.

X‐ray absorption spectroscopy (XAS), including X‐ray absorption near‐edge structure (XANES) and extended X‐ray absorption fine structure (EXAFS), was utilized to study the chemical state and coordination environment of Cu–C–N in Cu SACs. To determine the oxidation state of isolated Cu atoms in Cu SAC, the XANES spectra of Cu foil, CuO, and Cu SAC were measured (Figure 2e). The results show that the Cu K‐edge XANES characteristic peak of Cu SAC is located between Cu foil and CuO, indicating that the oxidation state of Cu is higher than metallic Cu⁰ but lower than Cu^2^⁺, consistent with the XPS results. In the Fourier‐transformed EXAFS spectra of the above samples in R‐space (Figure 2f; Figure S7, Supporting Information), no Cu–Cu characteristic peak (2.24 Å) was detected in Cu SAC samples.^[^ ^13c^ ^]^ Additionally, wavelet transform (WT) analysis of Cu K‐edge EXAFS oscillations was conducted to differentiate backscattering atoms (Figure 2g). The maximum WT intensity is at ≈7.3 Å^−^¹ in Cu foil and ≈5.8 Å^−^¹ in CuO sample, which are attributed to Cu–Cu and Cu–O, respectively. In contrast, the maximum WT intensity at ≈5.2 Å^−^¹ in Cu SAC‐BDC in K‐space is attributed to Cu–N structures, with no corresponding Cu–Cu or Cu–O intensity peaks. Based on HAADF‐STEM, PXRD, XPS, and XAS results, it can be confirmed that the as‐prepared Cu SACs are single‐atom nanomaterials. According to EXAFS fitting results (Table S1, Supporting Information), the quantitative structural parameters of Cu atoms in Cu SACs indicate that Cu atoms are coordinated with 3.6 ± 0.1 N atoms with an average bond length of 1.96±0.01 Å, reflecting a Cu‐N₄ coordination structure.

### 2.2 Enzyme‐Like Activity of Cu SAzyme

**Figure 3:**
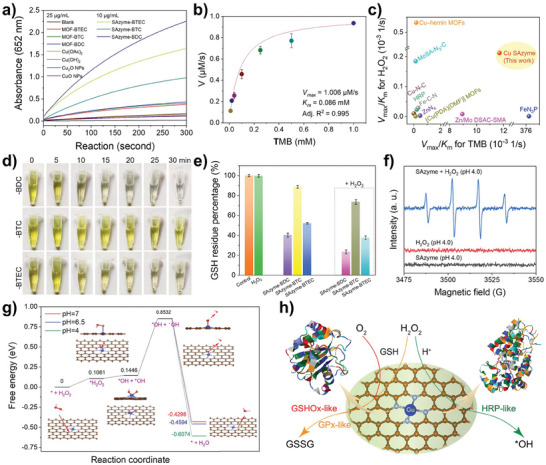
a) Comparison of HRP‐like activities of different copper materials. b) HRP‐like enzymatic kinetic curve of Cu SAzyme. c) Comparison of HRP‐like catalytic efficiency of Cu SAzyme with representative Cu‐based nanozymes.^[^ ^12^, ^15^, ^16^, ^29^ ^]^ d) Optical image of GSH oxidation catalysed by Cu SAzyme, e) Comparison of the GSH oxidation ability of Cu SAzyme in the presence or absence of H_2_O_2_. f) ESR spectra and g) DFT simulation of POD‐like reaction of Cu SAzyme. h) Schematic diagram of the multiple enzyme‐like reaction mechanisms of Cu SAzyme.

To investigate the POD‐like activity of SACs, common Cu‐based materials, including metal salts (copper hydroxide, copper acetate), metal oxides (copper oxide nanoparticles, cuprous oxide nanoparticles), and Cu‐MOFs, were compared with the three single‐atom nanozymes (SAzyme‐BDC, SAzyme‐BTC, and SAzyme‐BTEC) for HRP‐like activity. As shown in **Figure** **3**a, the HRP‐like catalytic activity of SAzyme was significantly superior to other Cu‐based materials, with SAzyme‐BDC exhibiting notably higher activity than SAzyme‐BTEC or SAzyme‐BTC. Therefore, SAzyme‐BDC was selected for subsequent studies. SAzyme alone could not oxidize TMB (Figure S8, Supporting Information), indicating specific H_2_O_2_‐related HRP‐like reactivity. Even at low H_2_O_2_ concentrations (10–100 µm), SAzyme showed significant H_2_O_2_ concentration‐dependent HRP‐like activity (Figure S9, Supporting Information). The HRP‐like activity of SAzyme increased with its concentration and was active at an ultra‐low concentration of 1 µg mL^−1^ (Figure S10a, Supporting Information). In contrast, in the temperature range of 10–60 °C, its activity was relatively stable and seemed to be temperature‐independent (Figure S10b, Supporting Information). These results pave the way for constructing SAzyme‐based bio‐nano cascade reaction platforms.

The HRP‐like activity of SAzyme was further studied by using the Michaelis‐Menten kinetics. As shown in Figure 3b and Figure S11 (Supporting Information), the kinetic fittings for TMB and H_2_O_2_ substrates had high correlation coefficients (*R* ^2^ > 0.969), indicating that SAzyme follows typical Michaelis‐Menten kinetics. The *V*_max_/*K*_m_ parameter was used to calculate and compare the POD catalytic efficiency of SAzyme with some reported Cu‐MOF nanozymes and single‐atom nanozymes (Figure 3c).^[^ ^12^, ^15^, ^16^, ^29^ ^]^ Although several nanozymes exhibited better reactivity than SAzyme for individual TMB or H_2_O_2_ substrates, SAzyme demonstrated superior catalytic efficiency for both TMB and H_2_O_2_ substrates overall, surpassing even natural HRP. In summary, SAzyme is an excellent single‐atom nanozyme with high HRP‐mimicking activity, suitable for constructing high‐activity cascade reaction antibacterial systems.

Overexpressed GSH can protect bacteria from oxidative damage caused by ROS produced by redox nanozymes. Nanozymes with GSH‐depleting functions are more efficient in antibacterial applications. Therefore, the catalytic oxidation activity of SAzyme for GSH was investigated. As shown in Figure 3d, using DTNB as the colorimetric indicator for GSH thiol groups, the degradation process of GSH catalyzed by SAzyme was observed. Within 30 minutes of the reaction, SAzyme catalyzed the degradation of GSH in the order of SAzyme‐BDC > SAzyme‐BTEC > SAzyme‐BTC, similar to the HRP‐like activity results, indicating the excellent pseudo‐enzyme properties of Cu‐N4 coordination. The corresponding spectral analysis also confirmed this trend (Figure 3e), and the introduction of low‐concentration H_2_O_2_ (100 µm) accelerated the GSH consumption rate. These results indicate that SAzyme exhibits both GSH oxidase (GSHOx) and glutathione peroxidase (GPx) activities. pH‐dependent experiments confirmed that SAzyme can exhibit dual HRP/GPx‐like activities over a wide pH range, with better performance in mildly acidic conditions (Figure S12, Supporting Information). Based on the HRP‐like and GSHOx/GPx‐like activity study results, SAzyme‐BDC was selected as the optimal single‐atom nanozyme for subsequent experiments.

### 2.3 Mechanistic Study of SAzyme Reactions

Fundamentally, HRP and GPx are both H_2_O_2_‐related peroxidases (POD‐like) involved in redox catalysis. ROS scavenger experiments confirmed that •OH is the primary ROS product in the HRP‐like reaction of SAzyme (Figure S13, Supporting Information). The characteristic signal of •OH was also observed in the electron paramagnetic resonance (ESR) spectra of the SAzyme and H_2_O_2_ co‐incubation system (Figure 3f), verifying the production of •OH during the POD‐like reaction of SAzyme. To better elucidate the HRP/GPx‐like activity of SAzyme, the POD‐like reaction mechanism was deduced using first‐principles density functional theory (DFT) calculations performed with the Vienna Ab initio Simulation Package (VASP) and the projector augmented wave (PAW) method. The pathways of the POD‐like reaction under different pH conditions were simulated.

(1) $$H_{2}O_{2}+*\to *H_{2}O_{2}$$

(2) $$*H_{2}O_{2}\to *\mathrm{OH}+*\mathrm{OH}$$

(3) ∗OH+∗OH→∗OH+•OH

(4) $$*\mathrm{OH}+H^{+}+e^{-}\to *+H_{2}O$$

where the asterisk (*) denotes the active site of the SAzyme.

As shown in Figure 3g, during the POD‐like process of SAzyme, H_2_O_2_ molecules are first adsorbed onto the Cu‐N_4_ active site and then uniformly dissociate to form two *OH groups. The Gibbs free energy barrier for these processes is only 0.1446 eV. Next, •OH radicals are generated on the active surface, which is the rate‐limiting step with a Gibbs free energy barrier of 0.7086 eV. Subsequently, the remaining *OH near the active center can adsorb H^+^ from the solution, forming surface‐adsorbed H_2_O molecules. This reaction is thermodynamically favorable and pH‐dependent. Finally, after the desorption of H_2_O molecules from the SAzyme surface, SAzyme returns to its initial state. Throughout the reaction, the negative Gibbs free energy (minimum −0.6074 eV) and relatively low apparent energy barrier (0.8532 eV) indicate that the POD‐like reaction catalyzed by the SAzyme is thermodynamically feasible. These theoretical calculations are consistent with the observed high POD activity of SAzyme. Therefore, the Cu‐N_4_ coordination active sites are revealed as the fundamental structure‐activity relationship principle of Cu SAzymes, which can not only mimic GSHOx to directly catalyze GSH oxidation but also can mimic HRP and GPx in the presence of H_2_O_2_ to produce •OH radicals and deplete more GSH (Figure 3h).

### 2.4 Construction and Nanozyme Activity of BioSAzyme

**Figure 4:**
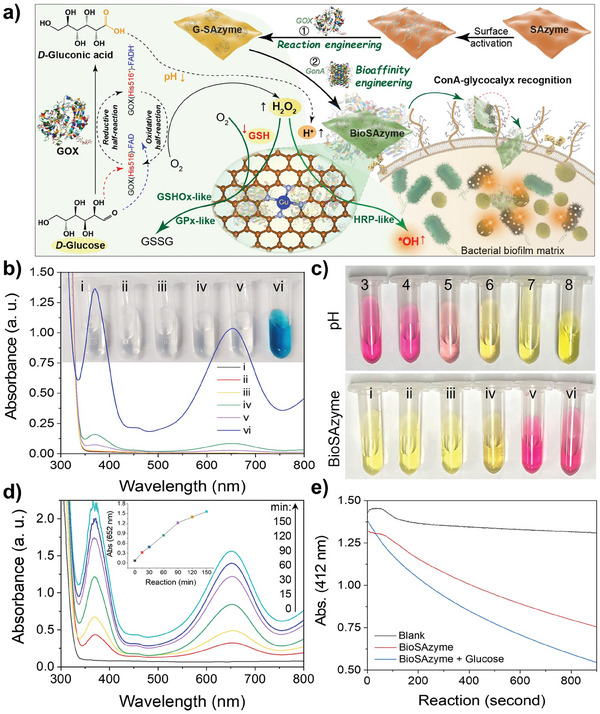
a) Schematic illustration of the synthesis and function of BioSAzyme reactor. b) HRP‐like activity of BioSAzyme, groups **i‐vi** refer to reaction systems of TMB in phosphate buffered saline (PBS) solution, glucose+TMB, GOX+TMB, BioSAzyme+TMB, GOX+glucose+TMB, or BioSAzyme+glucose+TMB, respectively. c) Methyl red pH determination of different pH standard solutions and BioSAzyme‐based reaction systems (i‐vi correspond to groups **i‐vi** in (b), respectively). d) Time‐dependent kinetics of HRP‐like BioSAzyme. e) Catalytic GSH oxidation by BioSAzyme.

To improve nanozyme‐based targeted antibiofilm therapy, a protein‐directed single‐atom bionanocatalytic nanoplatform with spatiotemporal precision was established by integrating the enzyme‐like properties of SAzyme, metabolic targeting of GOX, and glycocalyx bioaffinity of lectin ConA (**Figure** **4**a). GOX or GOX/ConA proteins were functionally immobilized on the surface of SAzyme to aggregate into G‐SAzyme and BioSAzyme, respectively. SEM images in Figure S14a,b (Supporting Information) indicate that protein engineering causes no significant morphological changes in SAzyme.

We further investigated the effect of protein modification on the catalytic activity of BioSAzyme. As shown in Figure 4b, glucose can trigger the cascade reaction of BioSAzyme (group iv), while colorimetric reaction observed in the control groups (i–v) was negligible. Moreover, it can be found that with the increase of GOX addition, the HRP‐like activity of BioSAzyme with H_2_O_2_ as substrate was significantly inhibited (Figure S15, Supporting Information), which may be the result of protein blocking Cu active site, and the glucose‐activated cascade reaction of BioSAzyme was preferred when 1.0 mg mL^−1^ of GOX was initially added. In this case, protein quantitative analysis (Figure S14c,d, Supporting Information) indicates that the protein loading rates of GOX and ConA in BioSAzyme are ≈10.12 and 16.67 wt.%, respectively.

Notably, the optimal activity conditions for HRP/GPx‐like SAzyme are mildly acidic pH (Figure S12, Supporting Information), which may limit its application in biofilms with dynamic pH environments.^[^ ^19c,d^ ^]^ However, the accumulation of gluconic acid during the GOX reaction may significantly adjust the pH microenvironment suitable for BioSAzyme. To verify this hypothesis, methyl red was used as a pH indicator to monitor the pH changes in the reaction solution (yellow at pH > 6.2, red at pH < 4.4). As shown in Figure 4c, compared with the standard pH solution group, the indicator was yellow in the control groups (i–iv), while a noticeable color change from yellow to red was observed in the GOX and glucose incubation group (v), indicating that the pH dropped from 7.4 to below 4.4. Similarly, after incubating BioSAzyme with glucose (vi), the indicator solution also turned bright red. Additionally, it was found that the pH change triggered by the cascade reaction of BioSAzyme and glucose is closely related to glucose concentration and reaction time (Figure S16a, Supporting Information). The pH changes in the BioSAzyme reactions were also determined using a pH meter. As shown, the pH values of reaction groups (i‐iv) remained slightly variable and reaction groups (v‐vi) decreased to around pH 4 (Figure S16b, Supporting Information). It can be also seen that the pH of the reaction system gradually decreased from pH 7.4 to about pH 3.6 within 3 h as the reaction time increased (Figure S16c, Supporting Information). Under the same conditions, as the glucose concentration increased, the pH value gradually decreased to below pH 4, while when the glucose concentration was higher than 2 mm, the pH value remained almost unchanged (Figure S16d, Supporting Information). Therefore, pH determination results are consistent with the colorimetric method, confirming the self‐regulatory effect of the pH environment in the BioSAzyme reaction.

Additionally, during the 150‐min continuous monitoring of the reaction between glucose and BioSAzyme (Figure 4d), it was observed that the HRP‐like activity of BioSAzyme increased due to the continuous accumulation of H_2_O_2_ and the steady decline in pH value. Similarly, in the presence of glucose, the GPx‐like reaction rate of BioSAzyme catalyzing the oxidation of GSH significantly accelerated (Figure 4e). These observations indicate that the generated gluconic acid enhances the multi‐enzyme catalytic activity of BioSAzyme. Furthermore, the characteristic ESR signal of •OH was detected in the co‐incubation system of BioSAzyme and glucose (Figure S17, Supporting Information). These results collectively demonstrate that endogenous molecules in biofilms (glucose, GSH) can effectively trigger the BioSAzyme bio‐nanoreactor.

### 2.5 Antibiofilm Activity and Mechanism of BioSAzyme

**Figure 5:**
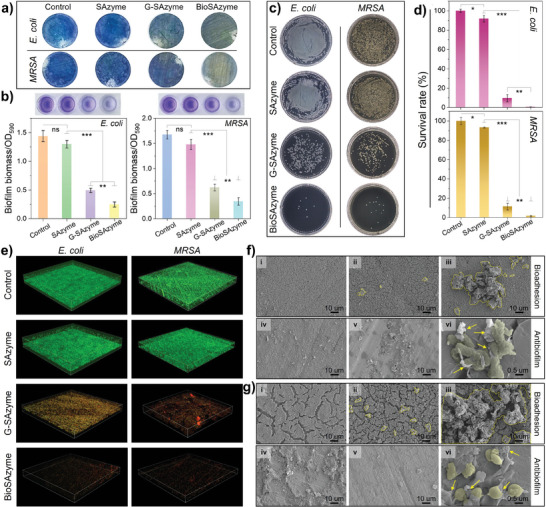
Crystal violet staining results of *MRSA* biofilms treated with PBS, SAzyme, G‐SAzyme, and BioSAzyme (from left to right): a) distribution of crystal violet on steel discs, b) color of ethanol elution solutions and the corresponding differences in biofilm biomass after different treatments. c) Culture plate results and corresponding d) bacterial viability statistics of bacteria within biofilms treated with PBS, SAzyme, G‐SAzyme, and BioSAzyme (from top to bottom), respectively. Data are presented as the mean ± SD. *n* = 3, ^*^ *p* < 0.05; ^**^ *p* < 0.01; ^***^ *p* < 0.001. e) Live/dead bacteria staining results of *E. coli* and *MRSA* biofilms treated with PBS, SAzyme, G‐SAzyme, and BioSAzyme groups, respectively. f–g) SEM images of *E. coli* and *MRSA* biofilm samples, respectively. In the SEM images, (i) refers to the untreated biofilm samples. (ii) and (iii) represent the adhesion results of G‐SAzyme and BioSAzyme to mature bacterial biofilms, respectively. (iv) and (v) represent the growth status of bacterial biofilms treated with G‐SAzyme and BioSAzyme, respectively. (vi) represents the close view of bacterial cells treated with BioSAzyme.

Given the strategic design, the antibiofilm efficacy of BioSAzyme was assessed using biofilm models of the Gram‐negative pathogen *Escherichia coli* O157: H7 (*E. coli* O157: H7) and the Gram‐positive drug‐resistant strain methicillin‐resistant *Staphylococcus aureus* (*MRSA*). Biofilms were cultured on sterile 304 steel surfaces and treated with nanozyme formulations during their growth phases. The biofilm extent and density were evaluated through crystal violet staining. Notably, in **Figure** **5**a, heavy staining was observed for the control and SAzyme‐treated groups, indicating substantial biofilm presence. In contrast, the surfaces treated with G‐SAzyme and BioSAzyme exhibited markedly reduced staining, signifying decreased biofilm biomass. Quantitative analysis from the ethanol elution of the stained biofilms (Figure 5b) showed that, while the SAzyme did not impact biofilm biomass significantly compared to the control group, both G‐SAzyme and BioSAzyme treatments notably reduced biofilm accumulation, with BioSAzyme achieving the most significant reduction. These outcomes suggest that BioSAzyme exhibited strong antibiofilm activity, followed by G‐SAzyme.

Colony counting from biofilm samples (Figure 5c) further supported these findings, indicating dense colony formation in controls and SAzyme treatments, while sparse colonies appeared after G‐SAzyme treatment, and very few were observed with BioSAzyme treatment, underscoring its potent biofilm inhibition. Similar trends were noted in *MRSA* biofilms, with significantly reduced bacterial survival in G‐SAzyme and BioSAzyme groups compared to the control. Figure 5d shows the corresponding colony count statistics. Compared with the control group, the bacterial survival rates in the *E. coli* biofilms were 91.81% (SAzyme), 9.64% (G‐SAzyme), and 0.36% (BioSAzyme), respectively. Correspondingly, the bacterial activities in the *MRSA* biofilms were 93.30% (SAzyme), 11.28% (G‐SAzyme), and 1.47% (BioSAzyme), respectively.

To elucidate the antibacterial mechanism, the survival status of biofilms was evaluated using confocal laser scanning microscopy (CLSM) with bacterial staining. As shown in Figure 5e, dense biofilm embedded with live bacteria aggregates was observed in the control group for both *E. coli* and *MRSA*. Similarly, SAzyme‐treated biofilms also exhibited vigorous growth. In contrast, after G‐SAzyme treatment, many damaged or dead bacterial cells were dispersed within the thin biofilm layers, indicating the poor growth status of *E. coli* and *MRSA* biofilms. Unlike other groups, only a few scattered dead bacterial components were found in the BioSAzyme‐treated samples, with almost no formed biofilm matrix observed. Thus, CLSM analysis results are consistent with crystal violet staining and plate counting results. In addition, in the in vitro antibacterial assay of planktonic bacteria, BioSAzyme also exhibited potent antibacterial activity against both *E. coli* and *MRSA* cells in the presence of glucose, as shown by the cell fluorescent staining results (Figure S18, Supporting Information).

Next, SEM was used to observe the adhesion of BioSAzyme to *E. coli* (Figure 5f) and *MRSA* (Figure 5g) biofilms on steel surfaces and the corresponding bacterial morphological changes during the anti‐biofilm treatment. The control group samples exhibited thick biofilm matrices (group i). During the observation of nanozyme‐biofilm bioaffinity interaction, only a small amount of G‐SAzyme (yellow loop line) remained attached to the biofilm (group ii) after washing, while a large amount of BioSAzyme (yellow loop line) adhered tightly to the biofilm surface due to the ConA‐glycocalyx binding (group iii). These results confirm that BioSAzyme can selectively localize and effectively adhere to bacterial biofilms for both *E. coli* and *MRSA*. Furthermore, SEM observation of biofilms treated with G‐SAzyme or BioSAzyme showed that the biofilms of *E. coli* and *MRSA* became significantly thinner with numerous holes (group iv), indicating that such bio‐cascade reactions effectively disrupted the survival environment of *E. coli* and *MRSA*. Moreover, after BioSAzyme treatment, only a few bacterial cells survived on the steel surface without forming any biofilm (group v), and these bacterial cells appeared deformed, wrinkled, and even ruptured, where bioadhesion of BioSAzyme (yellow arrow) to cells were also clearly observed. This evidence confirmed the selective recognition of BioSAzyme to *E. coli* and *MRSA* biofilms.

In summary, BioSAzyme not only recognized biofilms through specific lectin‐glycocalyx interactions but also responsively catalyzed the consumption of endogenous glucose and GSH, generating substantial ROS, which facilitated the spatiotemporally precise eradication of both *E. coli* and *MRSA* biofilms.

### 2.6 Antibiofilm Activity of BioSAzyme for Wound Healing

**Figure 6:**
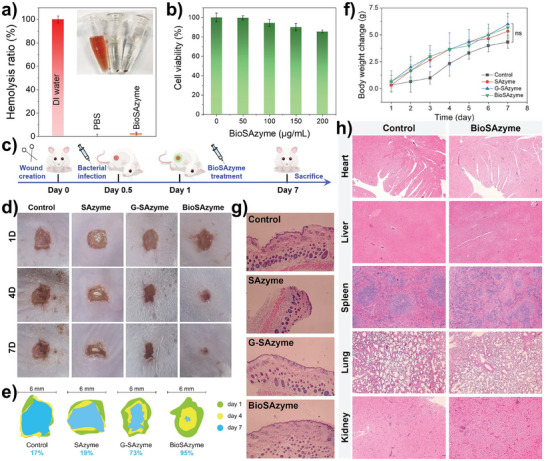
Evaluation of a) hemolysis and b) cytotoxicity of BioSAzyme. c) Schematic diagram of the BioSAzyme‐treated mice wound infection model. d) Optical images and e) morphology simulation of the wound areas. f) Body weight changes in mice. H&E staining images of g) infected wound epidermis and h) visceral tissue sections of mice after 7 days of different treatments. Data are presented as the mean ± SD. *n* = 3, ^*^ *p* < 0.05; ^**^ *p* < 0.01; ^***^ *p* < 0.001.

The hemolytic and cytotoxic properties of BioSAzyme were evaluated using fresh mouse red blood cells and NIH 3T3 cells as model cells, respectively. Hemolytic assessment results (**Figure** **6**a) showed that BioSAzyme exhibits potential blood compatibility, causing only 2.53% hemolysis in mouse red blood cells. As shown in Figure 6b, cell viability remained at 85.38% even with up to 200 µg mL^−1^ BioSAzyme, indicating minimal cytotoxicity. These findings suggest that BioSAzyme nanozymes have potential cell compatibility.

An in vivo model was constructed to assess the efficacy of BioSAzyme against *MRSA* biofilm infection in epidermal wounds in mice (Figure 6c). Kunming mice (4 weeks old) were wounded on their backs and injected with *MRSA* to induce infection. The mice were then randomly divided into four groups (control, SAzyme, G‐SAzyme, and BioSAzyme) and treated with the corresponding nanozyme formulations for wound dressing. As shown in Figure 6d, wounds in the control group healed slowly and showed signs of suppuration, similar to the SAzyme group. In contrast, wound healing was significantly accelerated in the G‐SAzyme and BioSAzyme groups. The time‐lapse simulation of wound shape changes (Figure 6e) indicated wound healing rates of ≈17%, 19%, 73%, and 95% for the control, SAzyme, G‐SAzyme, and BioSAzyme groups, respectively. The in vivo viable bacterial count results confirmed that BioSAzyme treatment showed the most effective antibacterial effect against *MRSA* infection compared with the other groups, with only 0.07% of *MRSA* viable cells near the wound area on day 7 (Figure S19, Supporting Information). These results demonstrate that BioSAzyme nanozymes exhibit excellent antibacterial effects in vivo through protein‐directed bio‐affinity adhesion and physiological responsiveness.

Additionally, body weight monitoring (Figure 6f) showed no significant differences between control mice and those treated with SAzyme, G‐SAzyme, or BioSAzyme. Furthermore, H&E staining of wound epidermal tissues (Figure 6g) revealed more complete epidermal layers and fewer inflammatory cells in the G‐SAzyme and BioSAzyme groups, indicating significantly improved epidermal healing compared to the control and SAzyme groups. Moreover, major organ tissue sections (Figure 6h) confirmed that BioSAzyme did not cause abnormal physiological or inflammatory damage in mice. These results collectively suggest that BioSAzyme can mediate self‐adaptive cascade reactions to effectively combat wound infections in mice without interfering with their physiological states, demonstrating potential safety for biofilm treatments.

## 3 Conclusion

In conclusion, we have designed a spatiotemporally guided copper single‐atom bionanozyme for precise antibiofilm treatments. Through mechanochemistry‐assisted pyrolysis, we synthesized a biomimetic N_4_‐coordinated copper single‐atom nanozyme with exceptional triple redox activities, including horseradish peroxidase‐like, glutathione peroxidase‐like, and glutathione oxidase‐like. Both experimental and DFT results indicate that the high peroxidase‐like activity stems from the N_4_‐coordinated single copper sites on the 2D nitrogen‐doped carbon structure. Surface engineering with 10.12 wt.% GOX and 16.67 wt.% ConA enabled the creation of BioSAzyme, which initiates spatiotemporally guided single‐atom bionanocatalysis targeting biofilm glycocalyx and metabolic environments. BioSAzyme demonstrated effective eradication of *E. coli* and *MRSA* biofilms in vitro, with bacterial survival rates of just 0.36% and 1.47%, respectively. Additionally, *MRSA* biofilm‐infected mouse wounds treated with BioSAzyme showed significant healing, with a 95% wound recovery rate and only 0.07% of live *MRSA* cells remaining on day 7. The antibiofilm mechanisms of BioSAzyme include 1) spatial recognition and penetration of biofilms via adhesive 2D nanostructures, 2) in vivo activation of single‐atom bio‐cascade reactions by endogenous glucose and the acidified pH microenvironment around biofilm, and 3) depletion of overexpressed GSH molecules in biofilm and generation of bactericidal ROS radicals for biofilm eradication. Future strategies in biofilm‐related therapies should focus on integrating protein functions with enzyme‐like single‐atom catalysis, emphasizing simple synthesis, cost‐effectiveness, and rational predesign of clinically applicable structure‐function correlations.

## 4 Experimental Section

Experimental information, including materials, characterizations, nanozyme synthesis, XAS data analysis, computational details, enzyme‐like catalytic assays, and in vitro and in vivo antibiofim experiments, are listed in the Supporting Information.

All animal experiments were conducted following the Guidelines of the Ministry of Health of the People's Republic of China (Document No. 55, 2001) and the Guide for the Care and Use of Laboratory Animals: Eighth Edition (ISBN‐10: 0‐309‐15396‐4). All experimental protocols were approved by the Animal Ethics Committee of Northwest A&F University (Shaanxi, China) (No. IACUC2024‐0604).

All results are expressed as mean ± standard deviation from at least three independent experiments. Statistical analysis was performed using Office Excel 2019 (Microsoft Corp., Redmond, USA). One‐way analysis of variance (ANOVA) was used to determine the differences between groups, with significance levels indicated by ^*^ *p* < 0.05, ^**^ *p *< 0.01, and ^***^ *p *< 0.001.

## Conflict of Interest

The authors declare no conflict of interest.

## Supporting information



Supporting Information

## Data Availability

The data that support the findings of this study are available in the supplementary material of this article.
