# Supplementary material for: Spatiotemporally Guided Single‐Atom Bionanozyme for Targeted Antibiofilm Treatment
Source: Small. 2024 Oct 6;20(51):2407747. doi: 10.1002/smll.202407747 (PMC11656685; doi:10.1002/smll.202407747)
Supplement: Supplementary file 1 — Supporting Information [file SMLL-20-2407747-s001.docx]

**Electronic Supporting Information for**

***Spatiotemporally Guided Single-Atom Bionanozyme for Targeted Antibiofilm Therapy***

**Lunjie Huang ^a, b, c^, Hongbin Pu ^a, b, c^, Da-Wen Sun ^a, b, c, d,^** ^^[[1]](#footnote-1)^*^

^a^ School of Food Science and Engineering, South China University of Technology, Guangzhou 510641, China

^b^ Academy of Contemporary Food Engineering, South China University of Technology, Guangzhou Higher Education Mega Center, Guangzhou 510006, China

^c^ Engineering and Technological Research Centre of Guangdong Province on Intelligent Sensing and Process Control of Cold Chain Foods, & Guangdong Province Engineering Laboratory for Intelligent Cold Chain Logistics Equipment for Agricultural Products, Guangzhou Higher Education Mega Centre, Guangzhou 510006, China

^d^ Food Refrigeration and Computerized Food Technology (FRCFT), Agriculture and Food Science Centre, University College Dublin, National University of Ireland, Belfield, Dublin 4, Ireland

**Experimental Section**

**1. Materials.** Copper(II) acetate anhydrous, dicyandiamide (DCD), terephthalic acid (H_2_BDC), trimesic acid (H_3_BTC), 1,2,4,5-benzenetetracarboxylic acid (H4BTEC), and glutaraldehyde were purchased from Macklin Biochemical (Shanghai, China). 3-Aminopropyltriethoxysilane (APTES), reduced glutathione (GSH), 5,5′-dithiobis-(2-nitrobenzoic acid) (DTNB), D-(+)-glucose (Glu), and methyl red were obtained from Aladdin BioChem (Shanghai, China). Hydrogen peroxide (30%) was sourced from Guanghua Sci-Tech (Guangdong, China). TMB and glucose oxidase (GOX, from *Aspergillus niger*) were acquired from Sigma-Aldrich (USA). Concanavalin A IV (ConA) and PI were purchased from Beijing Solarbio (China). The total protein assay kit (Bradford method) was obtained from Nanjing Jiancheng Bioengineering (China). SYTO-9 was purchased from Thermo Fisher Scientific (USA). LB broth and agar, tryptic soy broth (TSB), and PCA media were procured from Guangdong Huankai Microbial (China). Tris-HCl buffer and PBS powder were sourced from Shanghai Sangon (China). All reagents were of analytical grade. All aqueous solutions were prepared with deionized water (18.2 MΩ•cm, Millipore). Small steel discs (304 food-grade stainless steel, 1 cm in diameter) for biofilm culture were provided and processed by Guangzhou Daqiao Food Equipment Co., Ltd. (Guangzhou, China). The experimental strains, *E. coli* O157: H7 (ATCC 700728) and methicillin-resistant *Staphylococcus aureus* (*MRSA*, ATCC 43300), were purchased from Guangdong Engineering and Technology Research and Development Center of Microbial Food Safety (Guangzhou, China).

**2. Characterizations.** PXRD pattern was characterized by an X-ray diffractometer (Empyrean, PANalytical B.V., Netherlands) with a scan rate of 10° min^-1^. The SEM image was produced using a Zeiss Merlin FE-EM microscope (Carl Zeiss NTS GmbH, Germany). The HR-TEM image and the atomic-scale TEM image were collected with a JEM-2100Plus (JEOL, Japan) and a JEM-F200 (JEOL, Japan), respectively. The EDS maps were collected using a FEI Talos F200X G2 (Thermo Fisher Scientific Inc., USA) equipped with a Super-X G2 detector. The copper content in the SACs was determined by an iCAP 7200 Duo ICP-OES spectrometer (Thermo Fisher Scientific Inc., USA). XPS analysis was performed on an Axis Ultra DLD (Kratos Analytical Ltd., UK). UV-visible absorption data were recorded by a UV-1800 spectrophotometer (Shimadzu, Japan) or a Varioskan LUX microplate reader (Thermo Fisher Scientific, USA). Bacterial imaging was performed with a A1RMP two-photon confocal laser scanning microscopy (Nikon Instruments, Japan).

**3. X-ray absorption spectra (XAS) data analysis.** The XAS data, including XANES and EXAFS at the Cu K-edge of Cu SAC-BDC and reference samples, were conducted at the Singapore Synchrotron Light Source (SSLS) Center. The facility operated at 2.5 GeV with an average electron current below 200 mA using a pair of channel-cut Si (111) crystals in the monochromator. The XANES data at the Cu K-edge were collected in transmission mode. The obtained XAS data were processed using Athena (version 0.9.26) for background, pre-edge line, and post-edge line calibrations. Fourier transformed fitting was then performed in Artemis (version 0.9.26) ^[1]^. The fitting used k^3^ weighting, a k-range of 2.6-11 Å^-1^, and an R range of 1-3 Å. Four parameters were fitted: coordination number (C.N.), bond length (R), Debye-Waller factor (σ^2^), and E_0_ shift (ΔE_0_), without fixing, constraining, or correlating any of them. For Wavelet Transform analysis, the χ(k) exported from Athena was imported into the Hama Fortran code ^[2]^. The parameters were as follows: R range, 1-4 Å; k range, 0-13 Å^-1^; k weight, 2; and the Morlet function with κ=10, σ=1 was used as the mother wavelet to provide the overall distribution.

**4. Computational details.** First-principle density functional theory (DFT) calculations were performed using the Vienna Ab initio Simulation Package (VASP) with the projector augmented wave (PAW) method ^[3]^. The exchange-correlation functional was treated using the generalized gradient approximation (GGA) of the Perdew-Burke-Ernzerhof (PBE) functional ^[4]^. The energy cutoff for the plane wave basis expansion was set to 500 eV, and the convergence criterion for geometry relaxation was set to a force of less than 0.01 eV/Å on each atom. Grimme’s D3 correction was employed to account for dispersion interactions. The Cu SAC-BDC nanozyme model was constructed by doping the Cu-N4 moiety into the surface layer of a 5 × 5 graphene supercell with ~15 Å vacuum space added in the z-direction to avoid periodic interactions. The Brillouin zone integration was performed using 3 × 3 × 1 Monkhorst-Pack k-point sampling throughout all computational processes. The self-consistent calculations applied a convergence energy threshold of 10^-6^ eV.

**5. Preparation of Cu-MOFs.** Cu-MOFs were prepared using a simple mechanochemical grinding method. First, 1 mmol of copper(II) acetate anhydrous was weighed and added to an agate mortar, followed by the addition of 2 g of DCD. The mixture was ground evenly for 5 minutes. Subsequently, 1 mmol of the MOF ligand (H_2_BDC, H_3_BTC, or H_4_BTEC) was added to the mortar and ground for an additional 25 minutes. The resulting light blue powder samples were designated as DCD@Cu-MOFs. For comparison, an equal amount of copper(II) acetate anhydrous and MOF ligand were mixed and ground under similar conditions for 30 minutes to study the formation of Cu-MOFs. For SEM and PXRD characterization, the powders were washed several times with ethanol and dried at 60°C.

**6. Preparation of Cu SAzyme.** The preparation of SAzyme involves high-temperature annealing of DCD@Cu-MOF precursors in a nitrogen atmosphere using a quartz tube furnace. First, the DCD@Cu-MOF samples were placed in a crucible and heated to 800 °C at a rate of 3°C/min. The temperature was maintained for 3 h to complete the carbonization process. After allowing the samples to cool naturally to room temperature, they were soaked in a 5% hydrochloric acid solution and subjected to thorough etching under agitation for 4 h to remove possible aggregated metal/metal oxide particles. The samples were then washed with water until the pH was neutral. Finally, the black powder was vacuum-dried at 60 °C. The single-atom nanozyme products derived from DCD@Cu-BDC, DCD@Cu-BTC, and DCD@Cu-BTEC precursors were designated as SAzyme-BDC (or Cu SAC-BDC), SAzyme-BTC (or Cu SAC-BTC), and SAzyme-BTEC (or Cu SAC-BTEC), respectively.

**7. Preparation of G-SAzyme and BioSAzyme.** To modify protein molecules, we first chemically modified the SAzyme surface. In the first step, 20 mg of SAzyme was dispersed in 20 mL of ethanol solution (10%). After ultrasonic dispersion, 2 mL of APTES reagent was added while stirring. The mixture was stirred overnight at 60 °C. Finally, the sample was washed several times with water to remove excess APTES molecules, and the modified SAzyme was collected by centrifugation. **i)** To prepare GOX-modified G-SAzyme, the precipitate was resuspended in 2 mL of water, and 2 mL of glutaraldehyde (5%) was added. The mixture was stirred and incubated at 60 °C for 4 h. After washing several times and removing residual chemicals by centrifugation, the precipitate was resuspended in 5 mL of glucose oxidase (1 mg/mL) PBS solution (100 mM, pH 7.4) and ultrasonically dispersed at low power. The suspension was then incubated with shaking at 4 °C in the dark for 6 h. After the reaction, the precipitate and supernatant were collected by centrifugation. The precipitate was washed several times with PBS solution to obtain G-SAzyme, which was stored at 4°C. The protein content in the supernatant was determined using a protein assay kit (Bradford method). **ii)** To prepare GOX and ConA co-modified BioSAzyme, the obtained G-SAzyme was resuspended in 1 mL of water under low-power ultrasound, with 0.5 mL reserved. To the remaining 0.5 mL G-SAzyme solution, 2.5 mL of concanavalin A (1 mg/mL) PBS buffer was added, and the mixture was incubated with shaking at 4 °C in the dark for 6 h. After the reaction, the precipitate and supernatant were collected by centrifugation. The precipitate was washed several times with PBS solution to obtain BioSAzyme, which was stored at 4 °C. The protein content in the supernatant was also determined using a protein assay kit (Bradford method).

**8. Determination of HRP-like activity of SAzyme.** Typically, SAzyme (10 μg/mL) and other nanozymes (25 μg/mL) were added to a NaOAc buffer (20 mM, pH 4.0) containing TMB (0.5 mM) and H_2_O_2_ (10 mM). The HRP-like activity of these nanozymes was monitored over a fixed incubation period. The pH effect was measured likewise under different pH conditions (3.0-8.0). The HRP-like kinetics were monitored using the time-scan mode of the UV-Vis spectrophotometer at varying concentrations of TMB (0-1 mM) or H_2_O_2_ (0-50 mM). The enzymatic kinetic parameters were calculated according to the Michaelis-Menten equation (*v*=*V*_max_×[*S*]/(*K*_m_+[*S*])). All measurements were performed using a UV-1800 UV-Vis spectrophotometer or a microplate reader at 652 nm.

**9. Determination of GPx/GSHOx-like activity of SAzyme.** Firstly, SAzyme-BDC, SAzyme-BTC, and SAzyme-BTEC nanozymes (25 μg/mL) were incubated with GSH (1 mM) and H_2_O_2_ (100 μM) in Tris-HCl buffer (20 mM, pH 7.4) for 30 minutes. The mixture was then centrifuged, and DTNB (0.25 mM) was added to the supernatant and mixed thoroughly. The absorbance at 410 nm was measured, and photos of the reaction solutions were taken. To explore the effect of pH, the reactions were also conducted in buffers with different pH values. For time-dependent kinetic curves, SAzyme nanozymes (50 μg/mL), GSH (0.1 mM), DTNB (0.125 mM), and H_2_O_2_ (100 μM) were incubated together, and the change in absorbance at 410 nm was monitored using the time-scan mode of the UV-Vis spectrophotometer from the start of the reaction.

**10. Analysis of catalytic activity of BioSAzyme. i)** To analyze the GOX-HRP cascade catalytic activity of BioSAzyme, various PBS buffer solutions (0.5 mM, pH 7.4) containing glucose (5 mM), GOX (10 μg/mL), BioSAzyme (25 μg/mL based on SAzyme content), GOX+Glu, or BioSAzyme+Glu were incubated at 37 °C for 2 h. Afterward, TMB (0.5 mM) was added, incubated for 10 minutes, and the UV-Vis absorption spectra and solution colors were recorded. **ii)** To verify pH changes caused by gluconic acid production, the aforementioned reaction systems (PBS, Glu, GOX, BioSAzyme, GOX+Glu, or BioSAzyme+Glu) were incubated at 37 °C for 4 h and then incubated with 0.001% methyl red (ethanol solution). The color changes of the solutions were observed and recorded. For comparison, methyl red was mixed with standard pH solutions. Additionally, to study the relationship between reaction time and pH change, the Glu+BioSAzyme reaction system was incubated at 37 °C for various times (0, 0.5, 1, 2, 3 h), then 0.001% methyl red was added, and the color changes were recorded. Similarly, to study the relationship between glucose concentration and pH change, BioSAzyme was incubated with different glucose concentrations (0, 1, 2, 5, 10 mM) at 37 °C for 3 h, then 0.001% methyl red was added, and the color changes were recorded. The variations of pH value in these nanozynme reactions were also determined with a PB-10 digital pH meter (Sartorius Scientific Instrument, Germany). **iii)** To analyze the GOX-GPOx cascade catalytic activity of BioSAzyme, BioSAzyme (50 μg/mL), GSH (0.1 mM), DTNB (0.125 mM), and Glu (5 mM) were incubated together, and the change in absorbance at 410 nm was continuously monitored.

**11. Biofilm culture experiments.** A single colony of *E. coli* or *MRSA* was inoculated into 100 mL of LB broth and cultured at 37 °C with shaking until the bacterial suspension reached the log phase. Then, 10 μL of the bacterial suspension, adjusted to a final concentration of OD_600 nm_ = 0.01, was added to sterile 24-well cell culture plates containing 1 mL of TSB medium (0.25 wt% glucose) and sterile steel discs for biofilm growth. The plates were covered and sealed with sterile cling film and incubated statically at 37°C, with the medium replaced every 24 h.

**12. Antibacterial and antibiofilm activity study.** **i)** To study antibacterial activity, the planktonic bacterial suspension was centrifugated and washed with sterile isotonic saline solution (0.9% NaCl). Then, the planktonic bacteria (10^6 CFU/mL) were treated with nanozyme formulations (50 μg/mL BioSAzyme; or 50 μg/mL BioSAzyme + 10 mM glucose) in isotonic saline solution for 3 h. The bacterial sample was further stained with SYTO-9/PI to mark live and dead bacteria, and the bacterial survival was analysed by CLSM. **ii)** To study antibiofilm activity, sterile PBS or various nanozyme solutions (50 μg/mL) were added to the bacterial suspension in 24-well plates 12 h after the start of incubation in TSB medium. The plates were then incubated statically for an additional 36 h. After incubation, the steel discs were carefully removed with sterile tweezers and washed with sterile PBS to remove planktonic bacteria. PBS-treated samples served as controls. Biofilm activity was assessed using crystal violet staining and plate counting methods.

**13. Biofilm localization and morphology study. i)** To study the adhesion affinity of BioSAzyme to biofilms, mature *E. coli* and *MRSA* biofilm samples were incubated with the G-SAzyme or BioSAzyme in isotonic saline for several hours for sufficient recognition of biofilm matrix, and the samples were carefully washed several times with isotonic saline to remove randomly attached nanozymes. Samples were then prepared for SEM observation. **ii)** For morphological observation of the antibiofilm experiment samples, *E. coli* and *MRSA* biofilms grown in the presence of different nanozyme formulations were colloeted and prepared for SEM observation or further stained with SYTO-9/PI for CLSM analysis.

**14. Cytotoxicity assessment.** The cytotoxicity of the nanozymes was evaluated using the NIH 3T3 mouse embryonic fibroblast cell line as a model. Initially, frozen NIH 3T3 cells were cultured in 3T3 cell culture medium until they reached the logarithmic growth phase. Approximately 2×10^5 cells were then seeded into each well of a 96-well plate and incubated for 24 h. Subsequently, the cells were co-incubated with different types and concentrations of nanozymes for another 24 h. Cell viability was measured and calculated using the MTT assay.

**15. Hemolytic activity evaluation.** Fresh red blood cells obtained from mouse blood samples were used as the model cell. The blood samples were centrifuged to separate the red blood cells from the serum, washed three times with PBS buffer, and then resuspended in PBS buffer. The red blood cell samples were incubated in PBS, ultrapure water, and nanozyme solutions for 3 h. Finally, the suspensions were centrifuged, and the absorbance of the supernatant at OD492 nm was measured using a microplate reader to calculate the hemolysis rate.

**16. Mice wound infection model experiment.** Kunming mice (approximately four weeks old) were housed in cages under standard temperature (~25 °C) and humidity (60-70%) conditions for three days. After anesthetizing the mice, their dorsal hair was shaved, and a 6 mm radius wound was created using scissors. *MRSA* bacteria (20 μL, 10^8 CFU/mL) were then injected into the wound, and the mice were randomly divided into four groups (3-4 mice per group). After 12 h of *MRSA* infection, the mice were treated with different formulations (100 μL, pH 7.4) as follows: control, SAzyme, G-SAzyme, and BioSAzyme groups. The wound area changes were observed and recorded on days 1, 4, and 7. Mouse body weight was recorded daily. Viable bacterial counts near the wound tissue were performed on days 1 and 7. After seven days of treatment, the mice were sacrificed, and wound and major organ tissues were collected for H&E staining and histopathological analysis.

**Supplementary Figures and Tables**

**
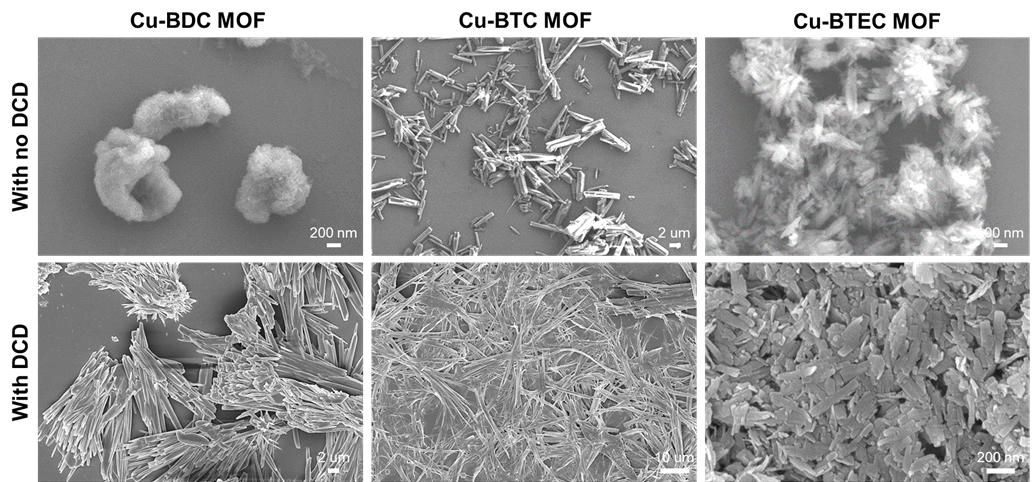
**

**Fig. S1.** SEM images of ligand-substituted Cu-MOFs and Cu-MOF@DCD prepared by mechanochemical synthesis.

***
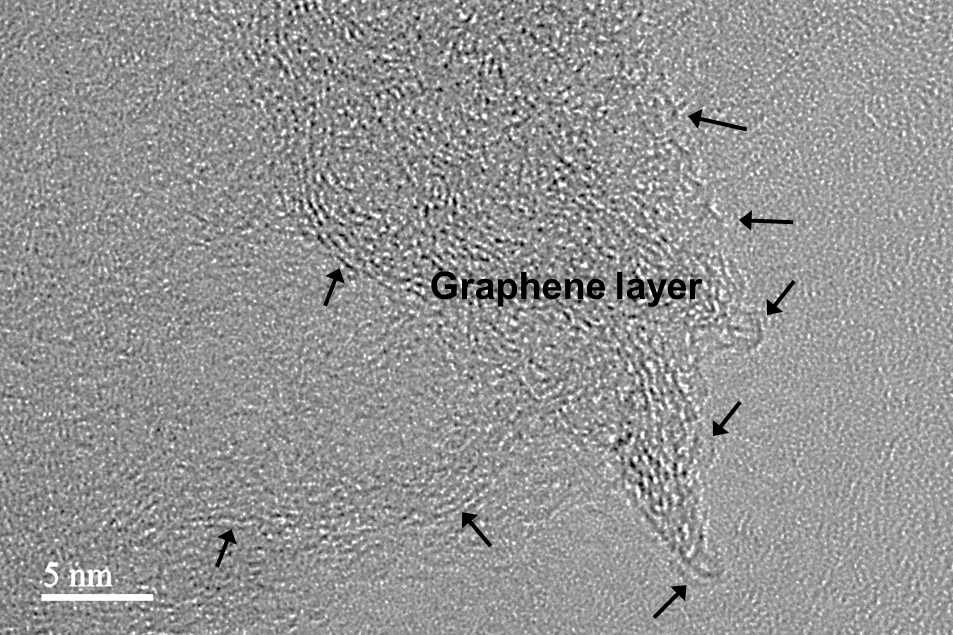
***

**Fig. S2.** HR-TEM image showing the thin carbon layer of Cu SAC nanosheet sample.

**Fig. S3.** PXRD pattern of the carbonization product of the DCD and H_2_BDC mixture.

**Fig. S4.** XRD patterns of the carbonization products of Cu-MOFs or the copper metal salt and DCD mixture.


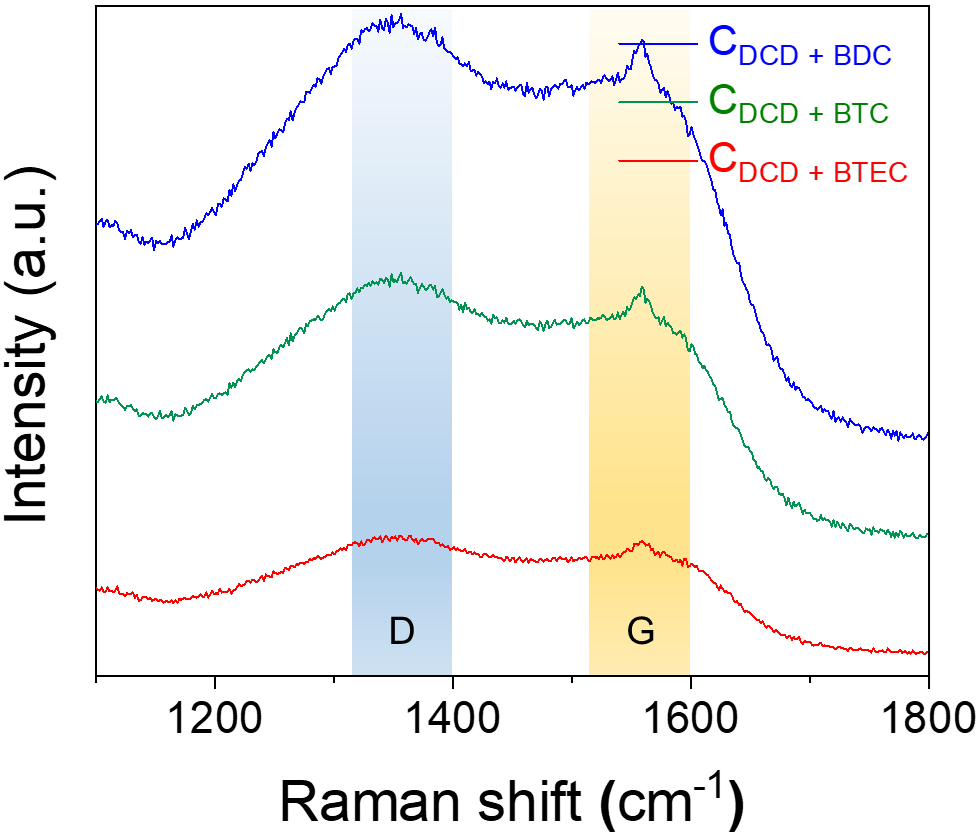


**Fig. S5.** Raman spectra of the carbonization products of mixtures of DCD and different MOF ligands.


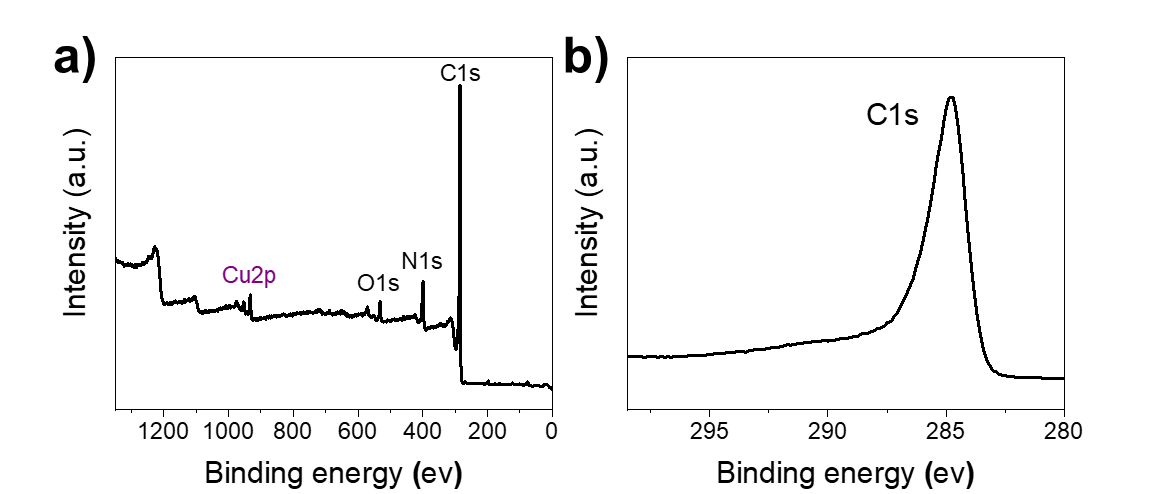


**Fig. S6.** XPS survey of Cu SACs. (a) Full spectrum and (b) C1s spectrum.

**
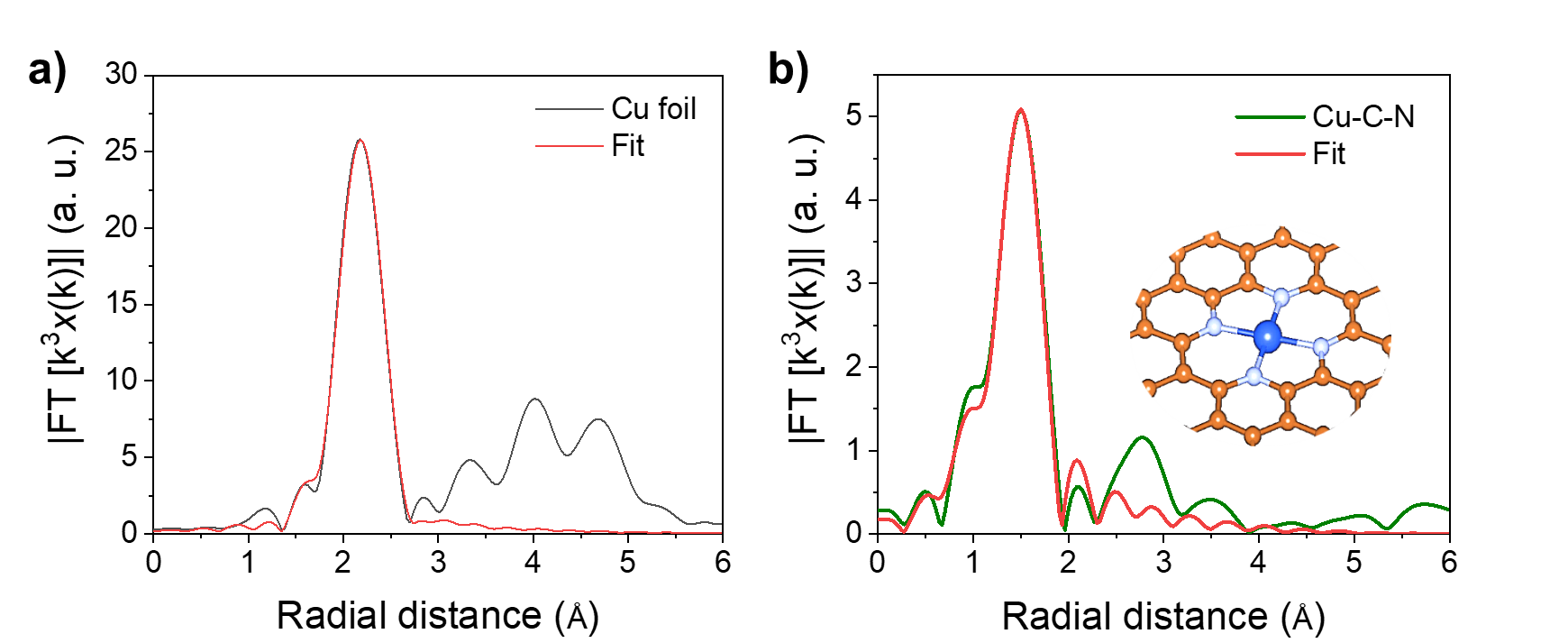
Fig. S7.** The EXAFS fitting curves for the samples a) Cu foil and b) Cu SAC. Inset is the proposed Cu-C-N architecture.

**
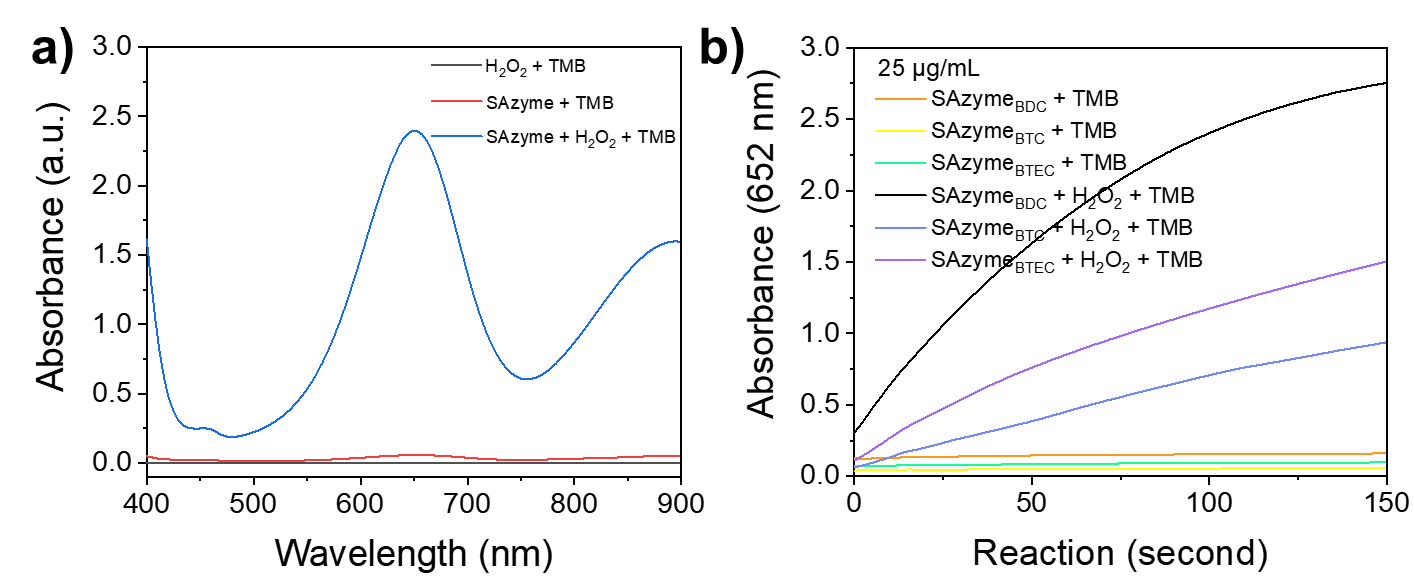
**

**Fig. S8.** Identification of oxidase-like or HRP-like activity of SAzyme. (a) UV-vis spectra for SAzyme-BDC, (b) Time-dependent activities for SAzymes.


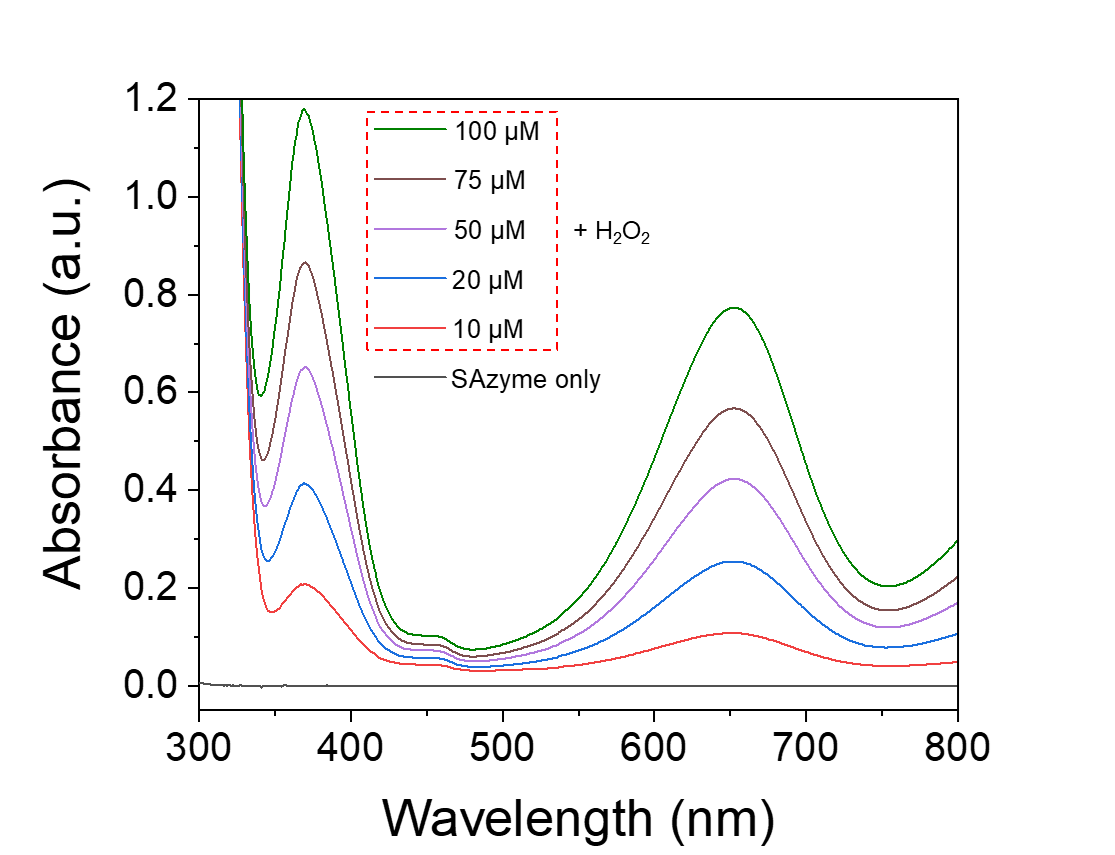


**Fig. S9.** H_2_O_2_ concentration dependence of HRP-like SAzyme.

*
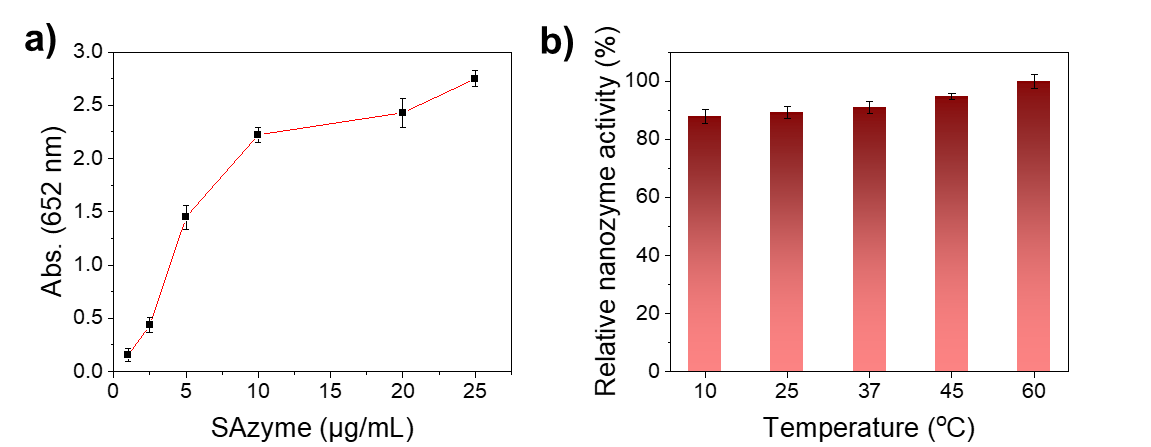
*

**Fig. S10.** Effects of (a) nanozyme concentration and (b) reaction temperature on the HRP-like activity of SAzyme.


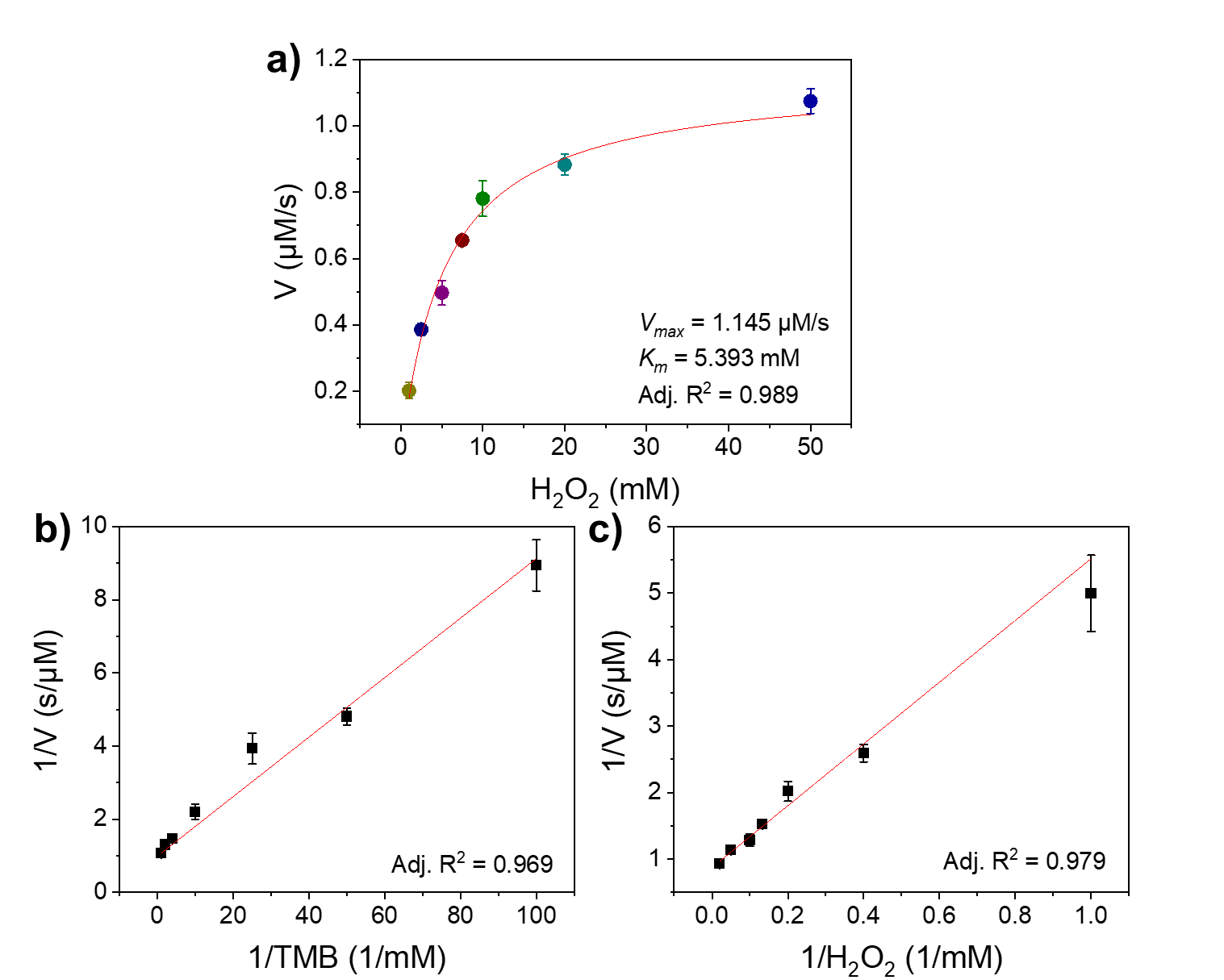


**Fig. S11.** Enzymatic kinetic profiles of HRP-like SAzyme. (a) kinetic curve for H_2_O_2_ substrate, and the corresponding (b-c) double-reciprocal fitting curves for TMB and H_2_O_2_, respectively.

**
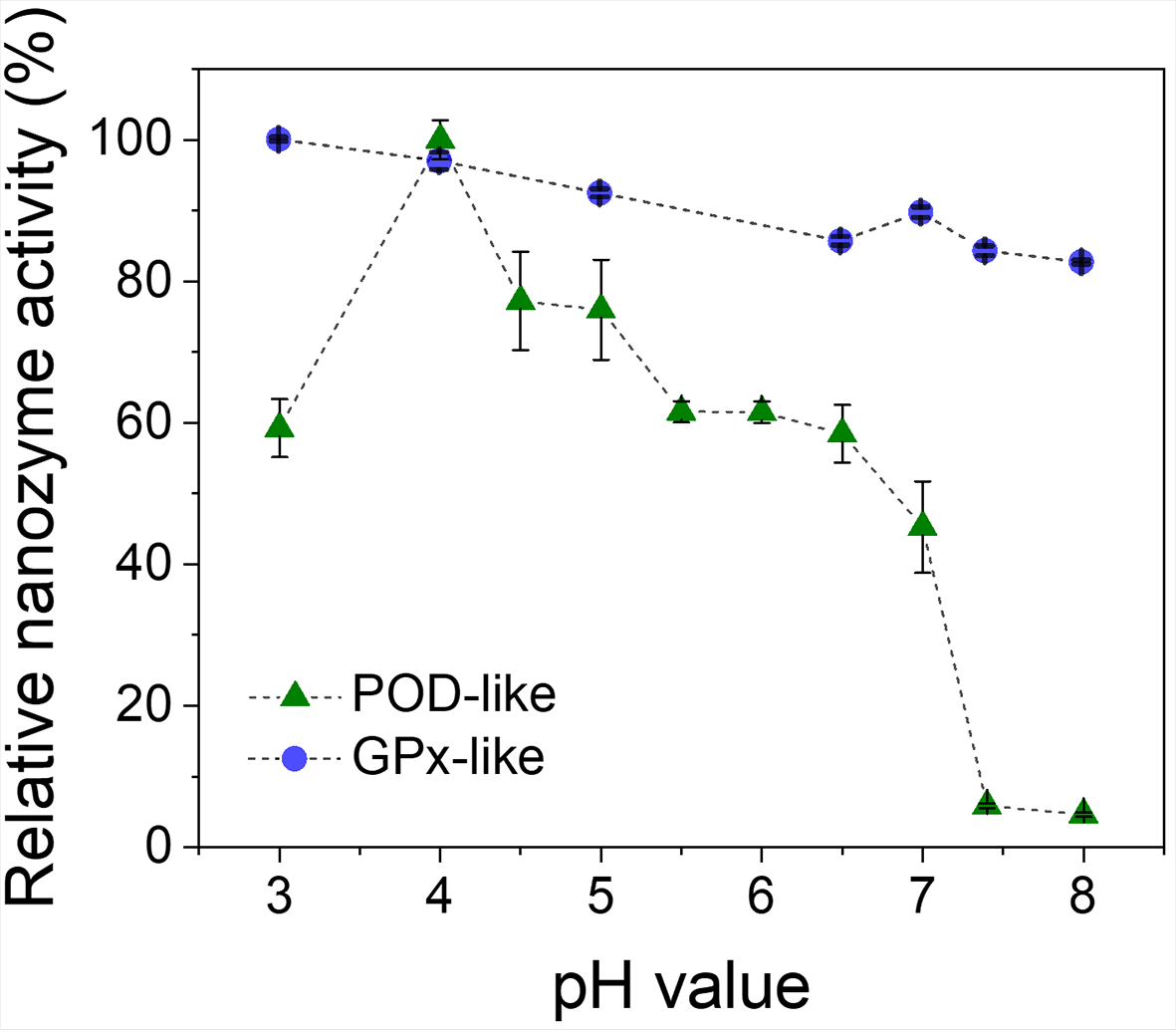
**

**Fig. S12.** Effects of pH conditions on the dual HRP/GPx -like activities of SAzyme.

**
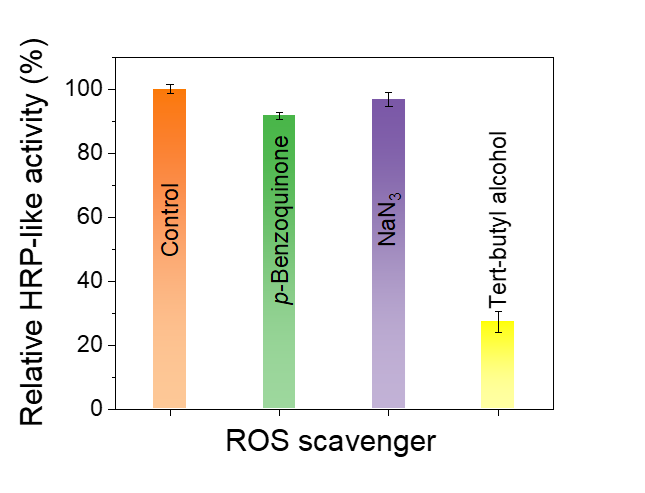
**

**Fig. S13.** The relative HRP-like activities of SAzyme in the presence of different ROS scavengers.

**
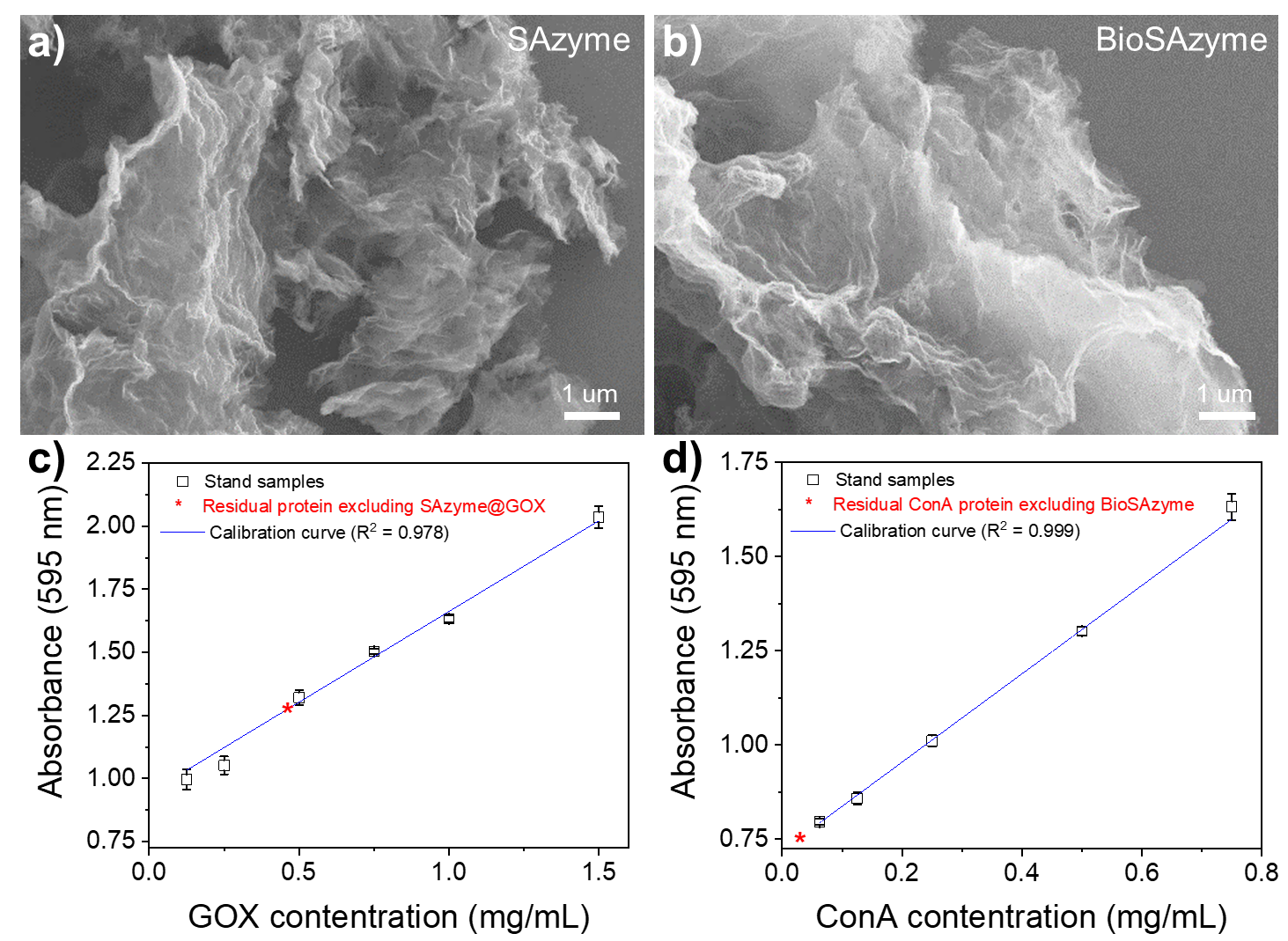
Fig. S14.** (a-b) SEM images of SAzyme before and after functionalization with GOX and ConA proteins. The linear relationships and results of protein quantification in the supernatant after (c) GOX modification or (d) ConA modification.


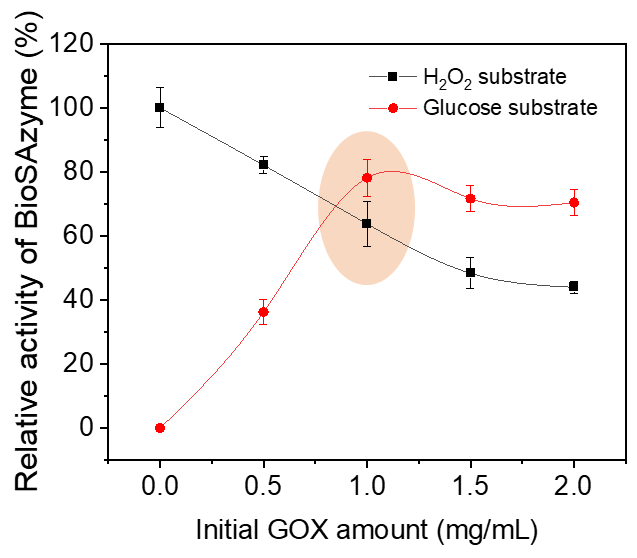


**Fig. S15.** Effect of initial GOX amount added on the enzyme-like activity of the resulting BioSAzyme.


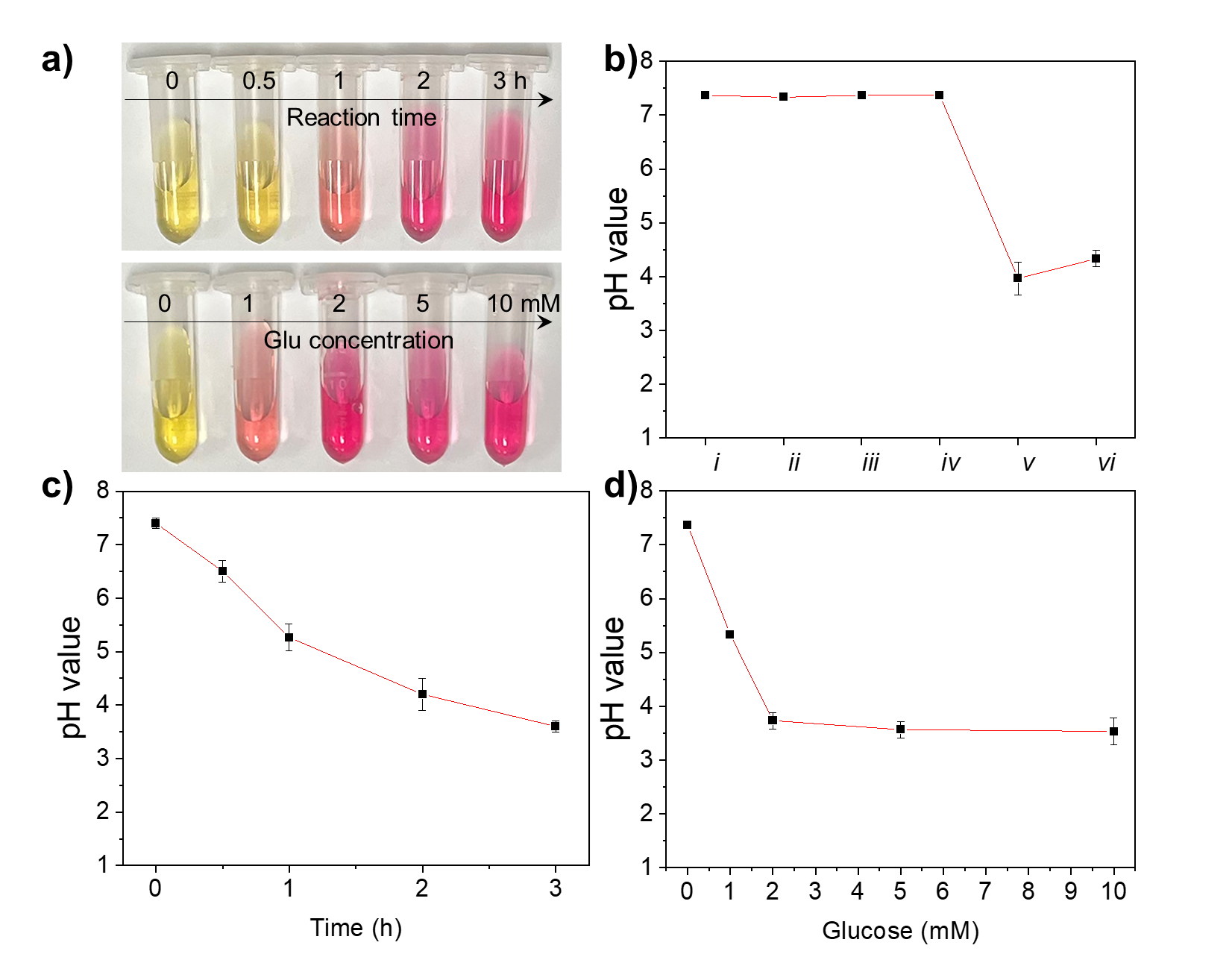


**Fig. S16.** (a) The methyl red colorimetric pH detection of BioSAzyme cascade reaction varied with reaction time and glucose concentration. Changes in pH value versus (b) reaction groups i-vi in Figure 4b-c, (c) reaction time, and (d) glucose concentration.

**Fig. S17.** ESR spectra of the HRP-like reaction of BioSAzyme.


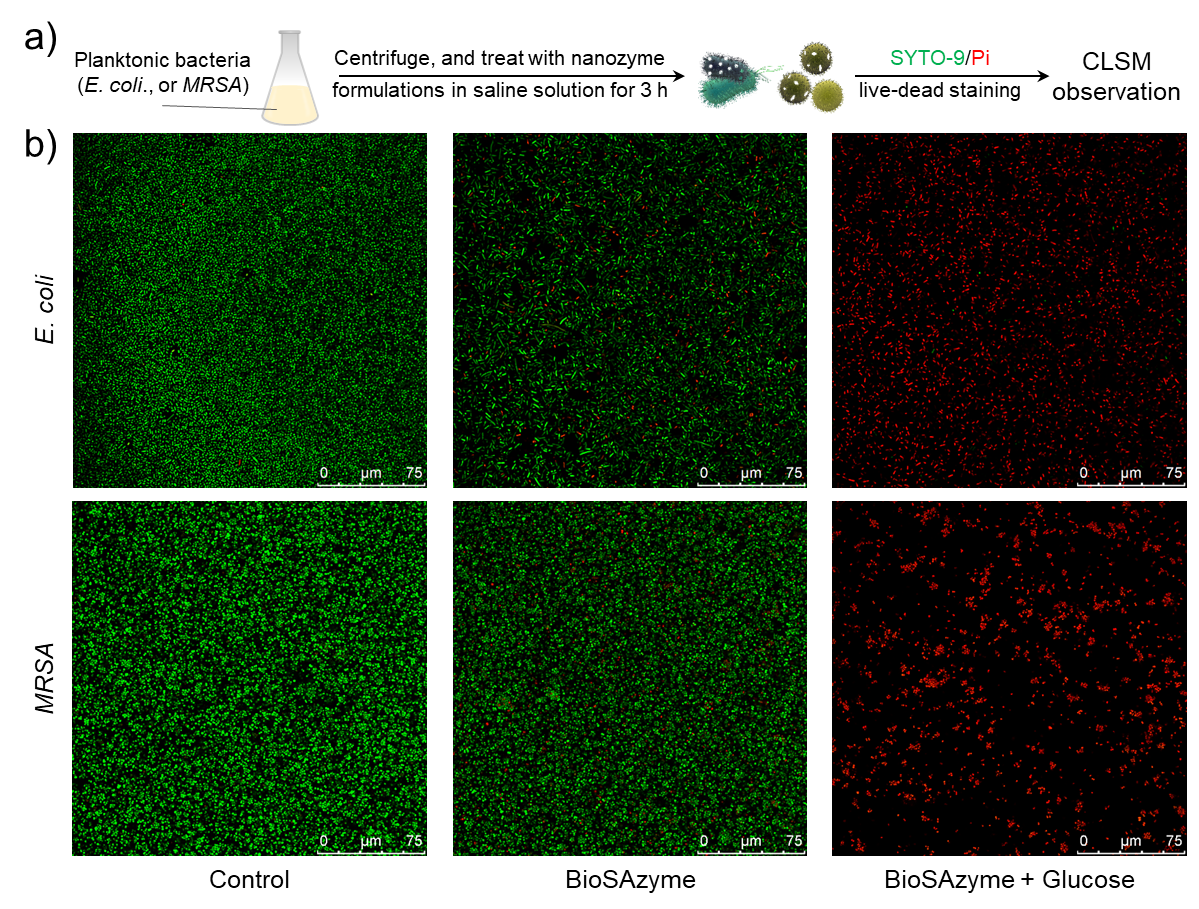


**Fig. S18.** (a) Procedure of *in vitro* antibacterial experiments on planktonic bacteria and (b) corresponding bacterial cell fluorescent staining images.


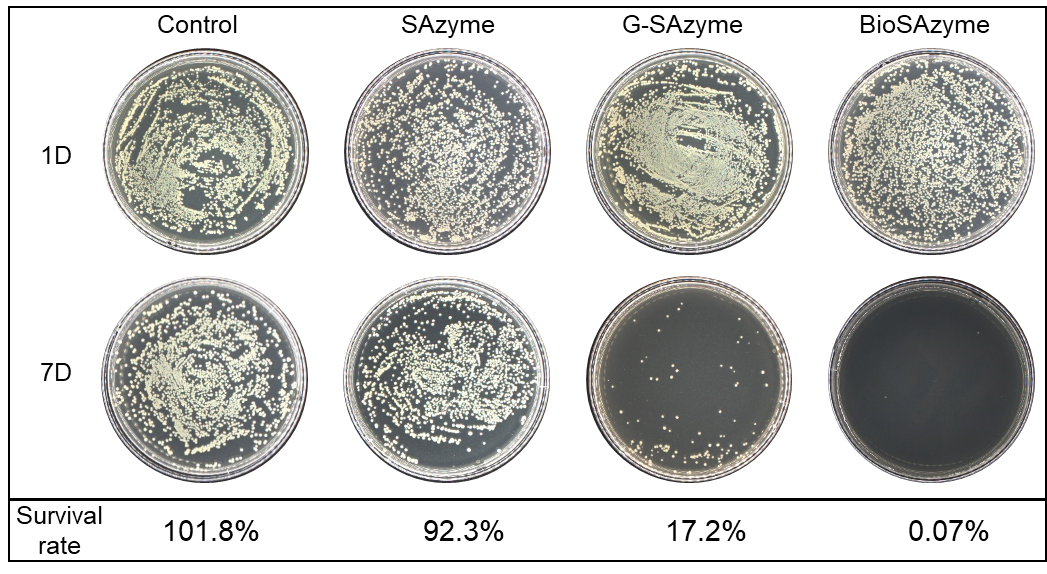


**Fig. S19.** Viable bacteria count near the wound tissue *in vivo*.

**Table S1.** Fitting parameters for Cu K-edge EXAFS.

| Sample | Path | C.N. | R (Å) | σ^2^ (Å^2^) | ΔE_0_ (eV) | R factor |
| --- | --- | --- | --- | --- | --- | --- |
| Cu foil | Cu-Cu | 12 | 2.54 ± 0.01 | 0.0083 | -7.1±1.0 | 0.0081 |
| Cu SAzyme | Cu-N | 3.6±0.1 | 1.96 ± 0.01 | 0.0058 | -2.4±1.0 | 0.0045 |

*^a^C.N.*: coordination numbers; *^b^R*: bond distance; *^c^σ*^2^: Debye-Waller factors; *^d^* Δ*E*_0_: the inner potential correction. *R* factor: goodness of fit. *Ѕ*_0_^2^ was set to 0.85, according to the experimental EXAFS fit of Cu foil reference by fixing C.N. as the known crystallographic value.

**Supplementary References**

[1] B. Ravel, & M. Newville. *Journal of Synchrotron Radiation* **2005**, 12(4), 537-541.

[2] H. Funke, A. C. Scheinost, & M. Chukalina. *Physical Review B* **2005**, 71(9), 094110; b) H. Funke, M. Chukalina, & A. C. Scheinost. *Journal of* *Synchrotron Radiation* **2007**, 14(5), 426-432.

[3] a) G. Kresse, J. J. P. r. B. Furthmüller, *Physical Review B* **1996**, 54, 11169; b) G. Kresse, J. J. C. m. s. Furthmüller, *Computational Materials Science* **1996**, 6, 15-50.

[4] J. P. Perdew, K. Burke, M. J. P. r. l. Ernzerhof, *Physical Review B* **1996**, 77, 865.

1. * E-mail: dawen.sun@ucd.ie; URLs: http://www.ucd.ie/refrig, http://www.ucd.ie/sun. [↑](#footnote-ref-1)
